# Diagnostic value of regional homogeneity and fractional amplitude of low-frequency fluctuations in the classification of schizophrenia and bipolar disorders

**DOI:** 10.1007/s00406-024-01838-4

**Published:** 2024-06-25

**Authors:** Giulia Cattarinussi, Fabio Di Camillo, David Antonio Grimaldi, Fabio Sambataro

**Affiliations:** 1 Department of Neuroscience (DNS), Padova Neuroscience Center (PNC), University of Padova, Azienda Ospedaliera di Padova, Via Giustiniani, 2, Padua, I-35128 Italy; 2 Padova Neuroscience Center, University of Padova, Padua, Italy; 3 Department of Psychological Medicine, Institute of Psychiatry, Psychology and Neuroscience, King’s College London, London, UK

**Keywords:** Functional magnetic resonance imaging, Machine learning, Psychosis support vector machine

## Abstract

Schizophrenia (SCZ) and bipolar disorders (BD) show significant neurobiological and clinical overlap. In this study, we wanted to identify indexes of intrinsic brain activity that could differentiate these disorders. We compared the diagnostic value of the fractional amplitude of low-frequency fluctuations (fALFF) and regional homogeneity (ReHo) estimated from resting-state functional magnetic resonance imaging in a support vector machine classification of 59 healthy controls (HC), 40 individuals with SCZ, and 43 individuals with BD type I. The best performance, measured by balanced accuracy (BAC) for binary classification relative to HC was achieved by a stacking model (87.4% and 90.6% for SCZ and BD, respectively), with ReHo performing better than fALFF, both in SCZ (86.2% vs. 79.4%) and BD (89.9% vs. 76.9%). BD were better differentiated from HC by fronto-temporal ReHo and striato-temporo-thalamic fALFF. SCZ were better classified from HC using fronto-temporal-cerebellar ReHo and insulo-tempo-parietal-cerebellar fALFF. In conclusion, we provided evidence of widespread aberrancies of spontaneous activity and local connectivity in SCZ and BD, demonstrating that ReHo features exhibited superior discriminatory power compared to fALFF and achieved higher classification accuracies. Our results support the complementarity of these measures in the classification of SCZ and BD and suggest the potential for multivariate integration to improve diagnostic precision.

**Supplementary Information:**

The online version contains supplementary material available at 10.1007/s00406-024-01838-4.

## Introduction

Schizophrenia (SCZ) and bipolar disorder (BD) present significant overlap in risk genes and clinical features [1, 2]. At the neuroanatomical level, they share a certain degree of ventricular enlargement and reduction in frontal lobe volume, while the temporal lobe is prominently involved in SCZ [3, 4]. Furthermore, the amygdala, hippocampus, and cingulate cortex show volumetric changes mainly in BD [5]. Structural connectivity is impaired at the frontal level in both disorders, while SCZ is associated with fronto-temporal deficits and BD with inter-hemispheric and limbic alterations [6]. Notably, the familiar risk for SCZ seems to be associated with cortico-striatal-thalamic alterations, while the risk for BD has been linked to changes in the cortico-striatal and limbic regions [7].

In recent decades, functional magnetic resonance imaging (fMRI) has allowed us to indirectly measure brain activity by detecting blood oxygen levels [8]. Besides its applications in task-based studies, fMRI can also be employed to explore neuronal activity during rest. Resting-state fMRI (rs-fMRI) is easily replicable and can be employed in those patients unable to complete long experiments, follow complex instructions, or perform cognitive tasks with a sufficient performance [9, 10]. The analysis of rs-fMRI data can provide a measure of the interaction between brain areas, the so-called functional connectivity (FC), which is defined as the statistical correlation between the activity of different areas [11]. In SCZ, rs-fMRI investigations have shown deficient FC within the prefrontal cortex (PFC) and between the cortical and subcortical regions, whereas, at the network level, alterations were found within the default mode network, the salience network, the executive network, and the sensorimotor network [12–15]. In BD, FC abnormalities have been reported mainly in the amygdala, cingulate cortex, and PFC [16]. More recently, novel methods have begun to be applied to fMRI signals to assess additional properties, such as regional activity and connectivity. Particularly, the amplitude of low-frequency fluctuations (ALFF) measures the intensity of spontaneous low-frequency signal fluctuations in the range of 0.01–0.08 Hz, which appears to contain physiologically meaningful information and to be related to FC [17]. Similarly, fractional ALFF (fALFF) represents the ratio of the power of each of those frequencies to that of the entire frequency range [18]. As a normalized index of ALFF, fALFF can provide a more specific measure of low-frequency oscillations. Furthermore, the fALFF measures seem to be less susceptible to gross pulsatile effects compared to ALFF [19]. In addition, regional homogeneity (ReHo) is a measure of localized FC, defined as the correlation between the time series of a particular voxel and those of neighboring ones [20]. Notably, a growing number of studies have shown that fALFF and ReHo are largely correlated and complementary in measuring local spontaneous activity [21–23]. In SCZ, there is evidence of altered ALFF/fALFF in the prefrontal, temporal, sensorimotor, striatal, and insular areas [24]. In addition, recent meta-analytic evidence has highlighted an increase in ReHo in the bilateral medial superior frontal gyrus, as well as a decrease in ReHo in the sensorimotor regions [25]. In BD, affective status seems to be associated with spontaneous brain activity and local connectivity changes, with studies showing a lower ReHo in depressed patients with BD in the frontal lobe, and altered fALFF in the superior and middle frontal gyrus, striatum, and cerebellum in individuals with mixed mood status compared to healthy subjects [26].

Recently, many studies have focused on improving the value of fMRI in clinical practice using pattern recognition and machine learning (ML) methodologies, harnessing the power of advanced algorithms to extract meaningful patterns and relationships from complex fMRI data (for a review, see Dwyer et al., 2018 [27]). In psychiatry, these approaches could help researchers uncover subtle brain biomarkers capable of distinguishing between different psychiatric conditions, thus improving diagnostic accuracy, informing treatment strategies, and advancing our understanding of the complex neurobiological underpinnings of mental health disorders [28].

In this study, we explored changes in brain activity in the resting state and local connectivity in a sample of patients with SCZ and BD and healthy controls (HC) and their correlations with clinical symptoms and cognitive performance. Our work had three main objectives: first, to better understand the shared and distinct neurobiological alterations of SCZ and BD; second, to evaluate the association between the features of rs-fMRI and clinical symptoms and cognitive functioning; third, to explore whether the alterations of rs-fMRI in SCZ and BD may present diagnostic validity. Based on previous literature, we hypothesized that spontaneous brain activity abnormalities would be identified in areas involved in the pathophysiology of SCZ and BD, including the prefrontal, striatal, and limbic areas, and that these would correlate with clinical symptoms and cognitive deficits. Furthermore, to corroborate these hypotheses, we applied ML to spontaneous brain activity to construct classifiers that could distinguish BD, SCZ, and HC and to identify the brain regions that contribute to this classification.

## Methods

### Participants

Patients with SCZ, BD, and HC were selected from the Consortium for Neuropsychiatric Phenomics dataset (https://openneuro.org/datasets/ds000030/versions/00016*).* The dataset contains data from 245 individuals with SCZ, BD, and HC, aged 21–50, with a minimum of eight years of formal education. All the procedures were approved by the Institutional Review Boards at UCLA and the Los Angeles County Department of Mental Health. Written informed consent was obtained from all participants. Subjects completed a magnetic resonance imaging (MRI) scan and a comprehensive neuropsychological battery. Exclusion criteria for the three groups were: (a) left-handedness; (b) a history of severe head injury with loss of consciousness; (c) contraindications to MRI. Moreover, HC were excluded if they had a lifetime or current diagnosis of any psychiatric disorder. From the initial sample, we excluded individuals with current medical illness, past or current substance use disorder, a history of major depressive disorder, anxiety disorders, and attention hyperactivity disorder. Three subjects did not complete the rs-fMRI acquisition and ten were excluded for excessive head-motion (see below). The final sample included 40 patients with SCZ, 43 patients with BD type I in partial or full remission, and 59 HC. Information on current psychiatric medications was available for all patients. The patients were on stable medication [29]. The dose of antipsychotics was transformed into chlorpromazine equivalents, while the dose of mood stabilizers and antidepressants was transformed into defined daily doses of drug intake (DDD) ratios, as described by the World Health Organization Collaborating Centre for Drug Statistics Methodology System of Defined Daily Doses.

### Clinical and cognitive assessment

Current and lifetime diagnoses were obtained with the Structural Clinical Interview for the Diagnostic and Statistical Manual of Mental Disorders, Fourth Edition (SCID) (DSM-IV). Psychopathological evaluation was carried out using the Hamilton Depression Scale (HAM-D) [30], the Scale for the Assessment of Negative Symptoms (SANS) [31], the Scale for the Assessment of Positive Symptoms (SAPS) [32], and the Young Mania Rating Scale (YMRS) [33]. Cognitive abilities were assessed with the Wechsler memory scale, California Verbal Learning test, Stroop test, Attentional network task, Continuous performance test, Task Switch task, and Stop Signal task.

### Imaging acquisition

Neuroimaging data were acquired on a 3 Tesla Siemens Trio scanner. A high-resolution T1-weighted anatomical scan (MPRAGE) was collected with the following parameters: slice thickness = 1 mm, 176 slices, TR = 1.9 s, TE = 2.26 ms, matrix = 256 × 256, FOV = 250 mm, sagittal plane. Functional MRI data were collected with a T2*-weighted echoplanar imaging (EPI) sequence with the following parameters: slice thickness = 4 mm, 34 slices, TR = 2 s, TE = 30 ms, flip angle = 90°, matrix = 64 × 64, FOV = 192 mm, oblique slice orientation. The rs-fMRI scan lasted 304 s. Participants were asked to remain still with their eyes open.

### Preprocessing

Structural and functional MRI data were pre-processed using Data Processing & Analysis for Brain Imaging (DPABI, http://rfmri.org/dpabi*)* and Statistical Parametrical Mapping 12 (SPM12) (http://www.fil.ion.ucl.ac.uk*)*, running under the MATLAB R2016a (The Mathworks, Sherborn, MA, USA). Briefly, all images were inspected for anatomical abnormalities or artefacts by an expert neuroimager (GC). The images were then reoriented and realigned for head motion correction. Subsequently, the T1-weighted images were segmented into grey matter (GM), white matter (WM), and cerebrospinal fluid (CSF). The mean WM and CSF signals were regressed from the time series to remove non-BOLD-related signals. Also, the 24-parameter motion regression proposed by Friston (Friston-24) was applied. GM images were then normalized to the Montreal Neurological Institute (MNI) space using DARTEL registration with a resulting isotropic voxel size of 3 mm x 3 mm x 3 mm. Ten subjects were excluded for excessive head motion.

### Spontaneous brain activity and local connectivity

To test the intrinsic neural activity differences between SCZ, BD, and HC, we estimated fALFF and ReHo using DPABI. For fALFF analyses, the filtered time series of each voxel was transformed into the frequency domain with a Fast Fourier Transform, and the power spectrum was obtained. First, we measured the square root of the signal across 0.01–0.08 Hz for each voxel [34]. Then, the sum of the amplitude values in the 0.01 to 0.08 Hz low-frequency power range was divided by the sum of the amplitudes over the entire detectable power spectrum (range: 0.01–0.1 Hz). The fALFF value for each voxel was z-normalized across the brain for each subject for standardization purposes. For ReHo, Kendall’s coefficient of concordance (KCC) was assigned to a given voxel by calculating the KCC of times series of the voxel and the nearest 26 neighboring voxels [20]. Higher ReHo values of a given voxel represent a higher degree of localized temporal synchronization within a neighboring cluster. Then, the ReHo value for each voxel was z-normalized across the entire brain for each subject to guarantee standardization. The fALFF and ReHo maps were smoothed with a Gaussian kernel of 4-mm full width at half maximum.

### Statistical analyses

The normality of the data was tested with the Shapiro-Wilk test. A principal component analysis with oblique Promax rotation was performed on cognitive scores to reduce the data to a smaller set of summary variables. Demographic, clinical, and cognitive data were compared using ANOVA, two-sample t-test, and χ^2^ test as appropriate. A one-way analysis of variance (ANOVA) was performed in SPM12 with the diagnostic group as a random effects factor on the whole brain fALFF and ReHo maps, respectively. Univariate t-tests were used to estimate pairwise diagnostic group differences. Two conjunction analyses were performed in SPM12 to identify overlap areas of significant differences in SCZ and BD relative to HC. The hypothesis of a conjunction contrast is true in those voxels where both individual contrasts are true. Thus, we tested for the common areas where both patients showed increased (SCZ and BD > HC: SCZ > HC ∩ BD > HC) and decreased (SCZ and BD < HC: SCZ < HC ∩ BD < HC) activity/connectivity, respectively. The voxel-wise threshold was set at *p* < 0.001 with the FWE cluster-level correction α = 0.05. Then, the smoothed and z-normalized fALFF and ReHo values were extracted from those regions that showed differences in three groups for post hoc analysis. The receiver operating characteristic curve (ROC) analysis was employed to assess the discriminative ability of abnormal responses in distinguishing individuals with SCZ and BD from HC using a support vector machine (SVM) for classification. This analysis aimed to identify specific brain regions with the highest diagnostic power among others.

Exploratory correlations were used to assess the relationship between fALFF and ReHo features for distinguishing patients from HC and clinical and cognitive scores. Furthermore, we performed exploratory correlation analyses between fALFF and ReHo values and chlorpromazine equivalents, mood stabilizers DDD, and antidepressants DDD. Statistical analyses were conducted with Jamovi (2.0.0.0). Statistical significance was assessed using an FDR α = 0.05.

### Classification analysis using SVM

SVM with linear kernel and L1-norm regularization was conducted using the tool NeuroMiner (version 1.1; https://www.pronia.eu/neurominer/) to investigate how the altered rs-fMRI features could discriminate SCZ and BD from HC. NeuroMiner is a ML software designed for translational research in neurology, psychology, and psychiatry that has been used in several peer-reviewed papers in these fields [35–37]. Although NeuroMiner is an open-source software, it requires MATLAB and does not encompass every types of advanced techniques for data imputation. Nonetheless, NeuroMiner does not require coding abilities and appears particularly suitable to implement the most widely used algorithm, such as SVM, which is broadly used in psychiatric neuroimaging studies [38]. In this study, we used the ReHo and fALFF features independently and in combination within a stacking model, an ensemble technique that synergistically integrates the predictive outputs of various models to enhance classification accuracy. Age and sex were regressed from ReHo and fALFF, and the residuals of the model were further scaled. We implemented a nested cross-validation, with 5-fold cross-validation with 10 permutations in the inner cycle and a leave-one-out cross-validation in the outer cycle. The parameter of the cost function C was optimized within the 5-fold cross-validation using a grid search among 11 parameters (as a geometric sequence, starting from 0.015625 and doubling each time, resulting in the following sequence: 0.015625, 0.03125, 0.0625, 0.125, 0.25, 0.5, 1, 2, 4, 8, 16). Finally, we evaluated the statistical significance of the classification results using a permutation test with 100 label iterations.

In addition, as previously described by Raio et al. [37], we compared the performance of the best classifier in assigning membership to HC or SCZ groups with those obtained by applying this classifier to BD and vice-versa to explore the generalization of the SCZ vs. HC features to BD and the BD vs. HC features to SCZ. To do so, we performed an out-of-sample cross-validation (OOCV) without any in-between retraining. Then, we employed a Kruskal-Wallis test to compare the OOCV-based decision scores for each of these groups.

Additionally, we conducted a classification analysis between SCZ and BD, utilizing both neuroimaging and clinical data (including HAMD, YMRS, SAPS, and SANS scores), independently and in combination within a stacking model, employing the same model setting described above.

## Results

### Demographic and cognitive data

The sample included 40 SCZ (mean age = 37.5 ± 8.6 years, 31 M), 43 BD type I (mean age = 35.1 ± 8.9 years, 26 M), and 59 HC (mean age = 33.1 ± 8.8 years, 32 M). No differences in age (F = 1.29, *p* = 0.28) and sex (χ^2^ = 5.64, *p* = 0.06) were observed between the groups. SCZ had higher SANS and SAPS scores compared to BD (*p* < 0.001), but no differences in HAM-D or YMRS were observed between the two groups (Table 1, Tab. S.1). The principal component analysis resulted in three components exploring memory (component 1), verbal learning and working memory (WM) (component 2), and inhibition (component 3) (see Tab. S.2 for details). SCZ had worse memory (component 1), verbal learning and WM (component 2) and higher inhibition (component 3) scores compared to BD and HC (all p’s < 0.001), while BD had lower memory (component 1) and verbal learning and WM (component 2) scores, and higher inhibition (component 3) scores compared to HC (all p’s < 0.046) (Tab. S.3).

**Table 1:** Clinico-demographic characteristics of the sample

	SCZ *n* = 40	BD *n* = 43	HC *n* = 59	statistics	*p*
*Demographics*					
Age m (SD)	37.5 (8.6)	35.1 (8.9)	33.1 (8.8)	F = 1.29	0.28
Sex M/F	31/9	26/17	32/27	χ2 = 5.64	0.06
*Clinical scales*					
HAM-D m (SD)	9.4 (7.9)	12.3 (8.8)	NA	U = 704	0.149
YMRS m (SD)	8.3 (7.1)	11.9 (10.7)	NA	U = 702	0.156
SANS m (SD)	7.1 (4.3)	2.3 (2.3)	NA	t=-6.26	< 0.01
SAPS m (SD)	9.2 (4.8)	5.1 (3.5)	NA	t=-4.44	< 0.01
*Current pharmacotherapy*					
Medications n (%)	37 (92.5%)	35 (81.4%)	NA	χ2 = 2.22	0.136
Antipsychotics n (%)	36 (90%)	22 (51.2%)	NA	χ2 = 14.9	< 0.001
CPZ equivalents mg	557 (624)	209 (278)	NA	U = 483	< 0.001
Mood stabilizers n (%)	10 (25%)	26 (60.5%)	NA	χ2 = 10.6	0.001
Mood Stabilizers dose (DDD)	0.146 (0.296)	0.693 (0.757)	NA	U = 516	< 0.001
Antidepressants n (%)	19 (47.5%)	15 (34.9%)	NA	χ2 = 1.36	0.243
Antidepressants dose (DDD)	0.671 (1.06)	0.520 (1.05)	NA	U = 748	0.246

BD: bipolar disorder; CPZ: chlorpromazine; DDD: defined daily doses; HAM-D: Hamilton Depression Scale; HC: healthy controls; m: mean; NA: not applicable: SANS: Scale for the Assessment of Negative Symptoms; SAPS: Scale for the Assessment of Positive Symptoms; SCZ: schizophrenia; SD: standard deviation; YMRS: Young Mania Rating Scale

### Group differences in fALFF

**Fig. 1:**
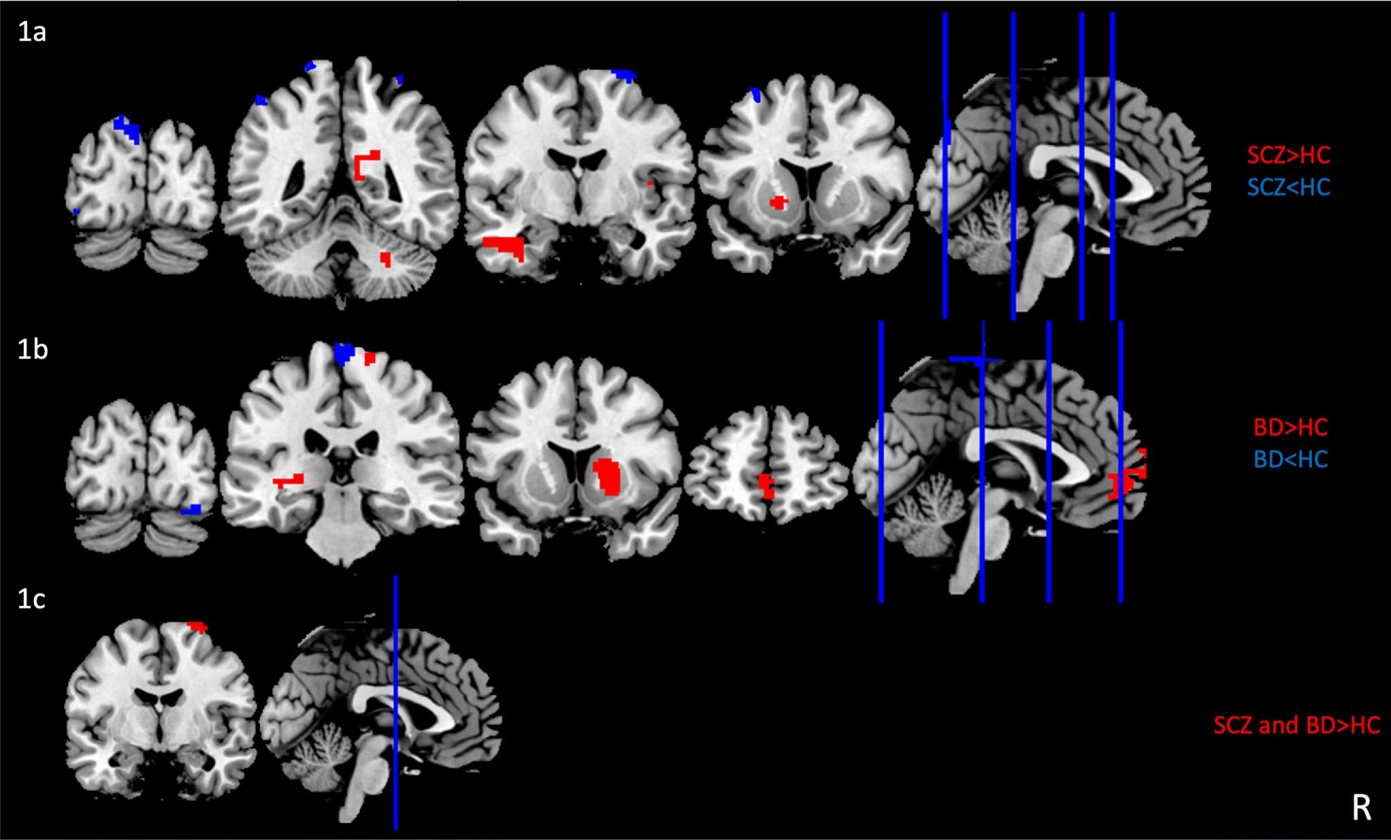
Group differences in spontaneous brain activity in individuals with SCZ and BD. Figure 1a**)** Brain areas with altered fALFF in SCZ compared to HC; Fig. 1b**)** Brain areas with altered fALFF in BD compared to HC; Fig. 1c**)** Brain areas with altered fALFF in SCZ and BD compared to HC. The significance level was set at a false discovery rate corrected *p* < 0.05. Red and blue denote increased and decreased fALFF values, respectively. R, right

*SCZ vs. HC.* We found a decrease in fALFF in SCZ compared to HC in the left middle frontal gyrus (MFG), right precentral gyrus, bilateral postcentral gyrus, bilateral superior parietal lobule (SPL), left supramarginal gyrus, left inferior occipital gyrus (IOG) and left cuneus. Conversely, SCZ presented an increase in fALFF in the right posterior cingulate cortex (PCC), the left inferior temporal gyrus (ITG), the right posterior insula, the left caudate, and the right cerebellum compared to HC (Fig. 1a).

*BD vs. HC.* BD showed lower fALFF compared to HC in the right middle postcentral gyrus, the right IOG, and the right occipital fusiform gyrus and higher fALFF in the bilateral MFG, the right precentral gyrus, the left transverse gyrus, the right caudate, and the left thalamus (Fig. 1b).

*SCZ vs. BD.* No significant differences in fALFF were found.

*SCZ and BD vs. HC.* The conjunction analysis showed higher fALFF in the right precentral gyrus in SCZ and BD relative to HC (Fig. 1c).

After correction for multiple comparisons, no correlations between fALFF values and medications were detected (Tab. S.4-S.6).

### Group differences in ReHo

**Fig. 2:**
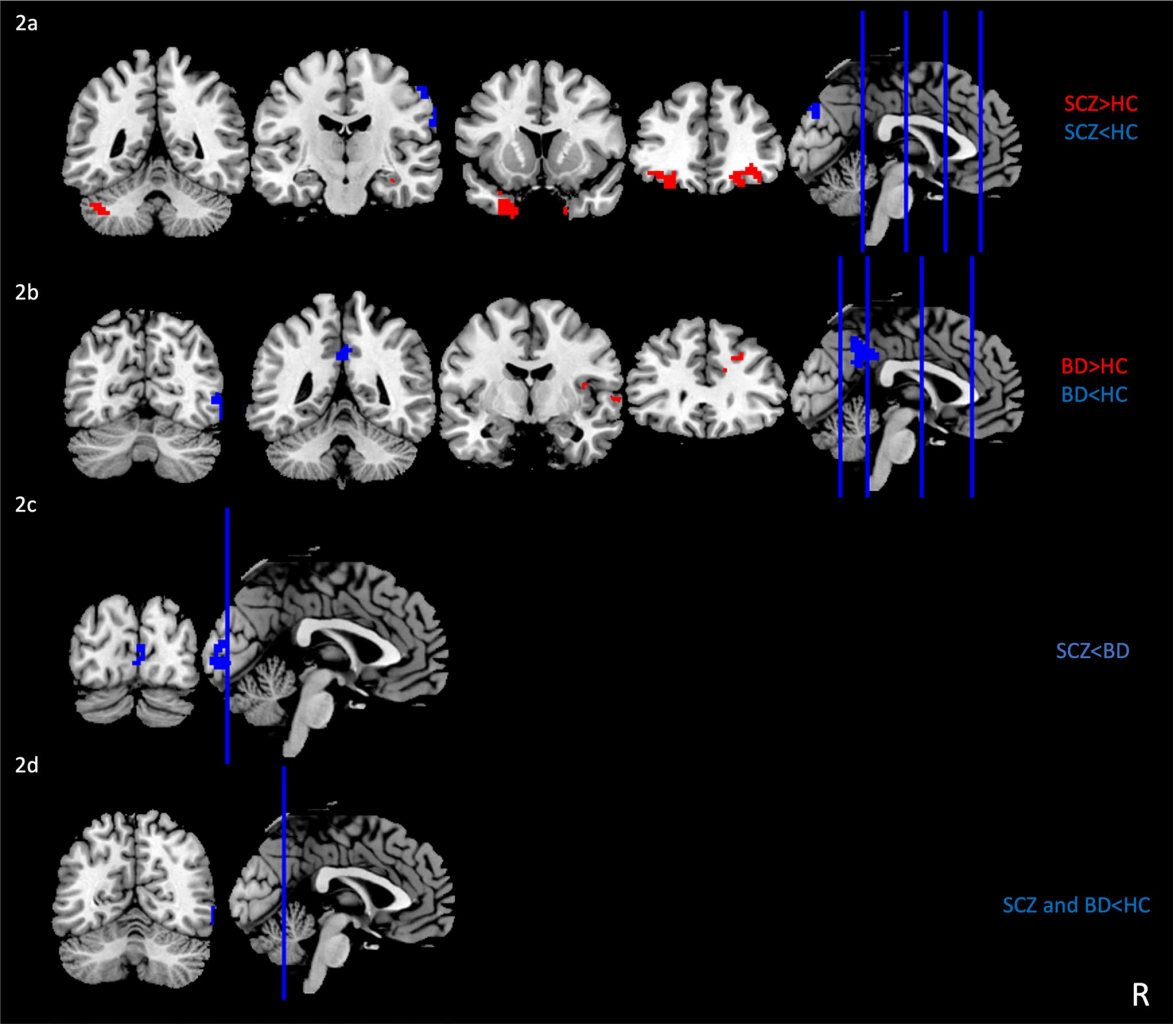
Group differences in local connectivity in individuals with SCZ and BD. Figure 2a) Brain areas with altered ReHo in SCZ compared to HC; Fig. 2b**)** Brain areas with altered ReHo in BD compared to HC; Fig. 2c**)** Brain areas with altered ReHo in SCZ compared to BD; Fig. 2d**)** Brain areas with altered ReHo in SCZ and BD compared to HC. The significance level was set at a false discovery rate corrected *p* < 0.05. Red and blue denote increased and decreased ReHo values, respectively. R, right

*SCZ vs. HC.* ReHo values were reduced in SCZ compared to HC in left SOG, right IOG and right postcentral gyrus, while increased in the right anterior orbital gyrus, left posterior orbital gyrus, bilateral temporal pole, right hippocampus, and left cerebellum (Fig. 2a).

*BD vs. HC.* In BD there was a decrease in ReHo compared to HC in the right middle temporal gyrus (MTG) and the left PCC and an increase in the right MFG, the right STG and the left anterior insula (Fig. 2b).

*SCZ vs. BD.* SCZ had lower ReHo compared to BD in the right calcarine scissure (Fig. 2c).

*SCZ and BD vs. HC.* The conjunction analysis highlighted a decrease in ReHo in the right IOG in SCZ and BD compared to HC (Fig. 2d).

After correction for multiple comparisons, we found no correlations between ReHo values and medications (Tab. S.4-S.6).

### Classification analysis using SVM

**Fig. 3:**
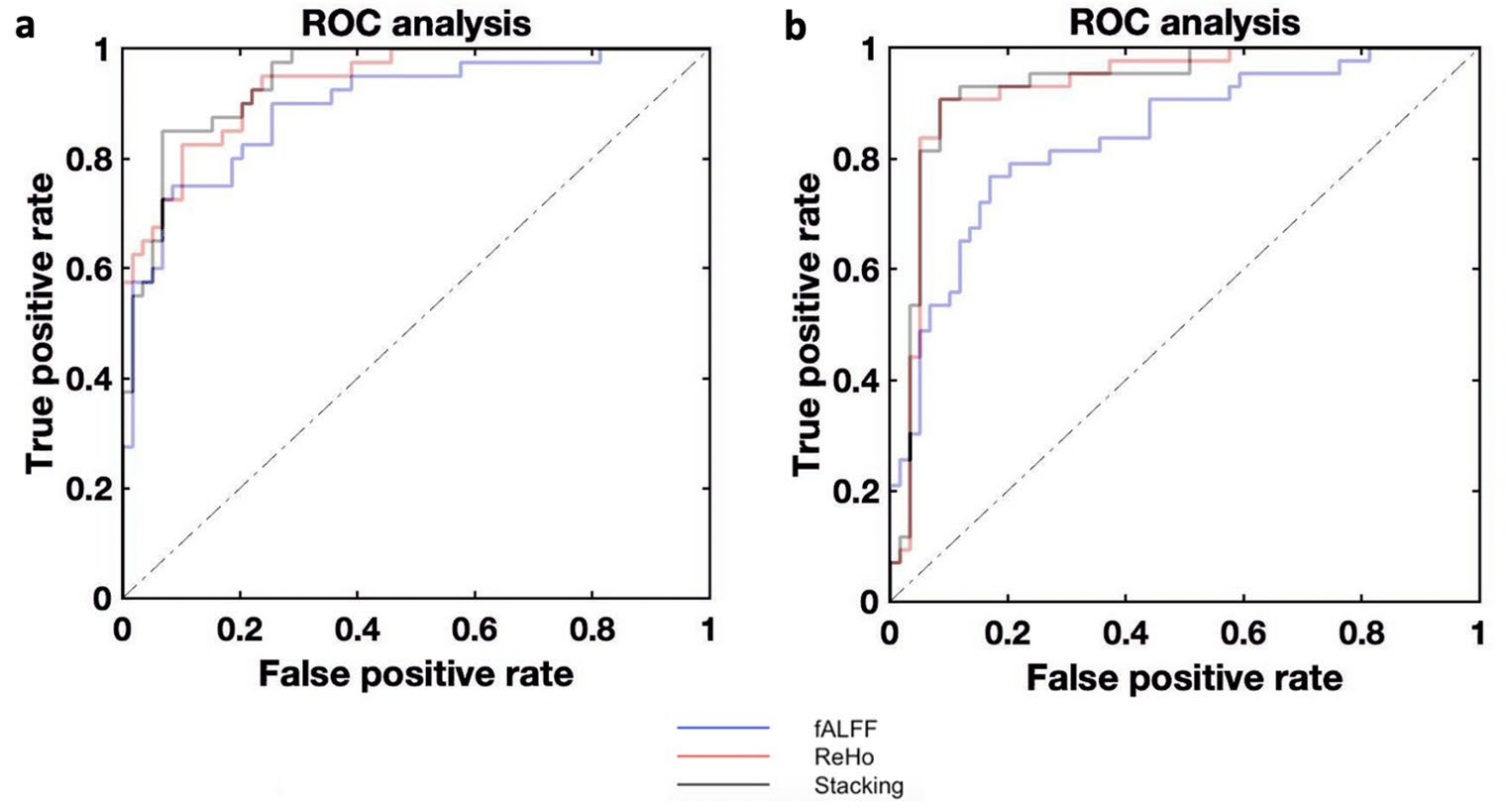
ROC curves of binary classification models. Figure 3a) ROC curve of SCZ vs. HC classification. Figure 3b) ROC curve of BD vs. HC classification. fALFF: fractional amplitude of low-frequency fluctuation; ReHo; regional homogeneity; ROC: receiver operating characteristic curve

*SCZ vs. HC.* The best performance was achieved using the stacking model for SCZ vs. HC (BAC: 87.4%, *p* < 0.01, Fig. 3a; Table 2). ReHo had a better discrimination performance compared to fALFF (BAC: 86.2% vs. 79.4%, *p* < 0.01). The most important features were the ReHo values of the right hippocampus, right temporal pole, left posterior orbital gyrus, left cerebellum, and right anterior orbital gyrus, and the fALFF values of the left ITG, left SMG, right cerebellum, bilateral SPL, and right posterior insula.

*BD vs. HC.* The best performance was achieved using the stacking model (BAC: 90.6%, *p* < 0.01, Fig. 3b; Table 2). ReHo showed a better discrimination performance compared to fALFF in both analyses (BAC: 89.9% vs. 76.9%, *p* < 0.01). The most important features were the ReHo values of the right MFG, the right STG, and the left anterior insula, and the fALFF values of the right caudate, the left transverse temporal gyrus, and the left thalamus.

**Fig. 4:**
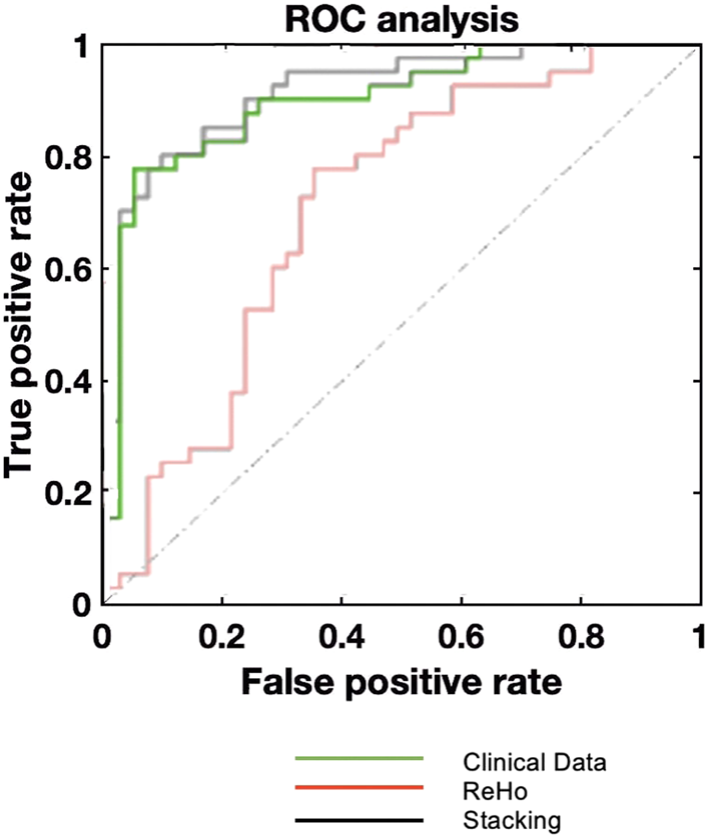
ROC curve of SCZ vs. BD classification. ReHo; regional homogeneity; ROC: receiver operating characteristic curve

*SCZ vs. BD.*  The best performance was achieved using the clinical and stacking model (BAC: 84.1%, *p* < 0.01, Fig. 4; Table 2). Clinical data demonstrated greater discrimination performance compared to ReHo (BAC: 84.1% and 70.1%, respectively; all *p* < 0.01). The most important features were SAPS (bizarre behavior, delusions, and hallucinations) and HAMD scores, and the ReHo value of the right calcarine scissure.

**Table 2:** Classification analysis using SVM

	SE	SP	BAC	AUC
**SCZ vs. HC**				
ReHo	82.5%	89.8%	86.2%	0.94 (0.88–0.99)
fALFF	77.5%	81.4%	79.4%	0.90 (0.83–0.97)
Stacking	85.0%	89.8%	87.4%	0.94 (0.88-1.00)
**BD vs. HC**				
ReHo	88.4%	91.5%	89.9%	0.93 (0.87–0.98)
fALFF	67.4%	86.4%	76.9%	0.84 (0.76–0.92)
Stacking	93.0%	88.1%	90.6%	0.93 (0.88–0.99)
**SCZ vs. BD **				
ReHo	77.5%	62.8%	70.1%	0.71 (0.60–0.82)
Clinical data	77.5%	90.7%	84.1%	0.90 (0.83–0.97)
Stacking	77.5%	90.7%	84.1%	0.92 (0.86–0.98)

AUC: area under the curve; BAC: balanced accuracy; BD: bipolar disorder; HC: healthy controls; SCZ: schizophrenia; SE: sensitivity; SP: specificity

**Fig. 5:**
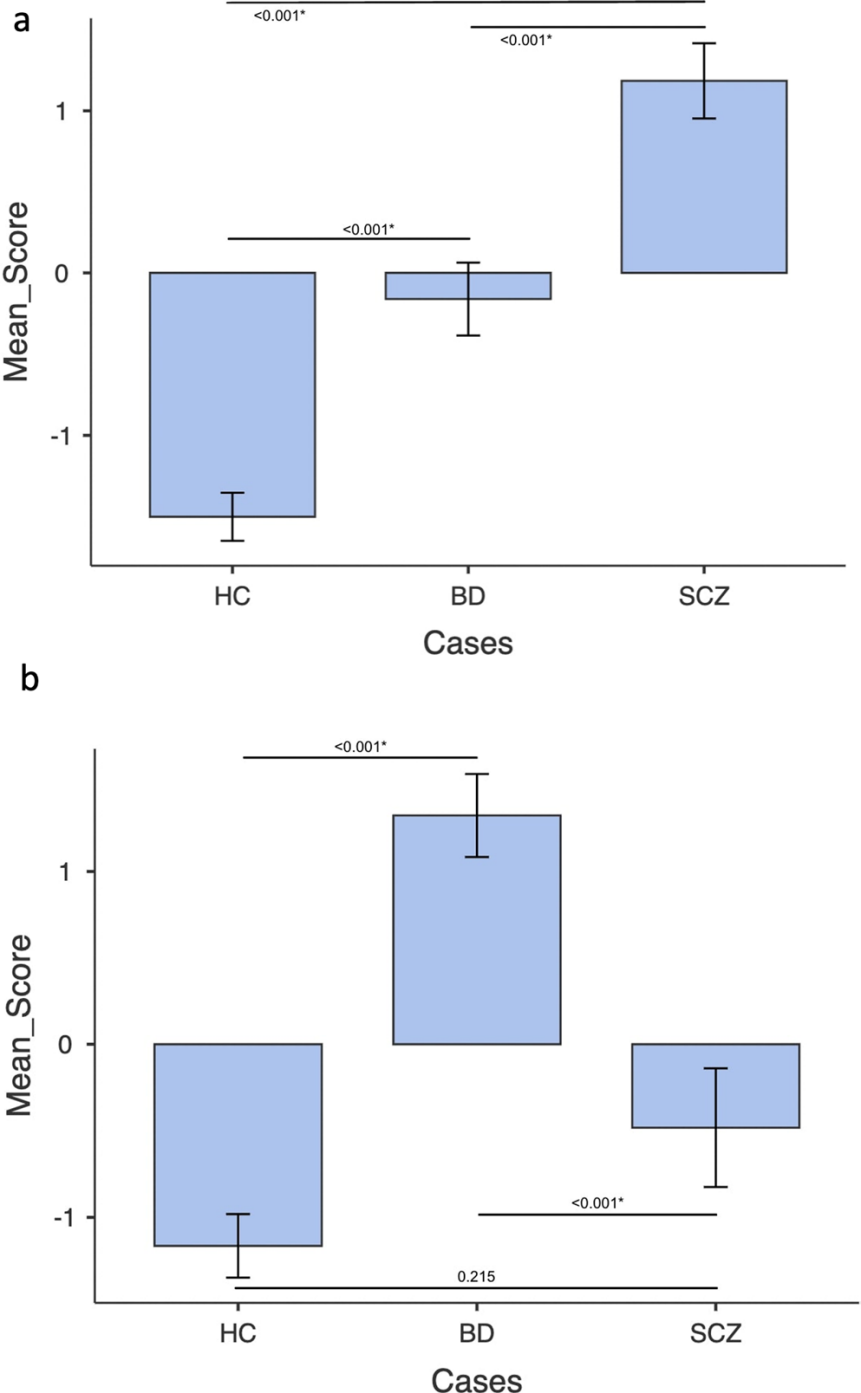
Application of classifiers discriminating between HC and SCZ to BD and classifiers discriminating between HC and BD to SCZ. Kruskal Wallis tests were conducted to compare decision scores from the stacking-based models discriminating HC vs. SCZ patients (Fig. 5a) and HC vs. BD patients (Fig. 5b) after the OOCV procedure. The error bars represent the standard error

*Out of sample validation.* The classifiers discriminating between HC and SCZ had a discriminatory value for BD (all *p* < 0.001) relative to HC and SCZ, while the classifiers discriminating between HC and BD had not a discriminatory value for SCZ (*p* *<* 0.001 and *p* = 0.215) (Fig. 5).

### Association between discriminatory fALFF and ReHo features and clinical symptoms and cognitive deficits in SCZ and BD

**Fig. 6:**
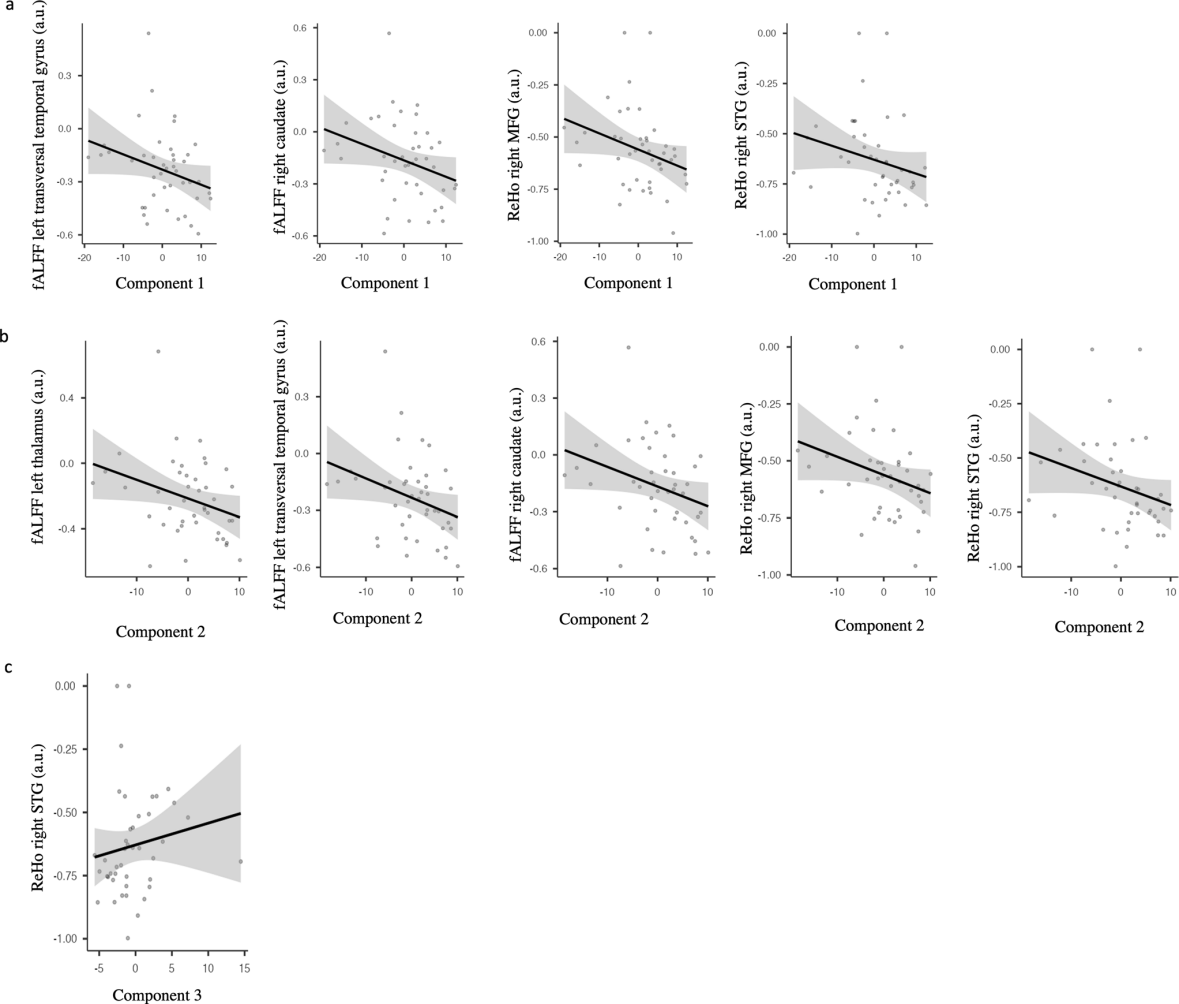
Scatterplot of the correlations between cognitive factors and fALFF and ReHo values in BD. Figure 6a) Scatterplot of the correlations between component 1 (memory) and fALFF and ReHo values in BD; Fig. 6b**)** Scatterplot of the correlations between component 2 (verbal learning and WM) and fALFF and ReHo values in BD; Fig. 6c**)** Scatterplot of the correlations between component 3 (inhibition) and fALFF and ReHo values in BD. fALFF: fractional amplitude of low-frequency fluctuation; MFG: middle frontal gyrus; ReHo; regional homogeneity; STG; superior temporal gyrus. All measures are shown in arbitrary units (a.u.)

Exploratory analyses showed that, in BD, component 1 (memory) was negatively correlated with the fALFF values of the left transverse temporal gyrus (rho=-0.316 *p* = 0.04), and the right caudate (rho=-0.336 *p* = 0.028), as well as with the ReHo values of the right MFG (rho=-0.390 *p* = 0.01) and the right STG (rho=-0.364 *p* = 0.016) (Fig. 6a). Component 2 (WM) was negatively correlated with the fALFF values of the left thalamus (rho=-0.312 *p* = 0.042), left transverse temporal gyrus (rho=-0.361 *p* = 0.018), and the right caudate (rho=-0.359 *p* = 0.018), and the ReHo values of the right MFG (rho=-0.355 *p* = 0.019) and the right STG (rho=-0.379 *p* = 0.012) (Fig. 6b). Component 3 (inhibition) was positively correlated with the ReHo values of the right STG (rho=-0.303 *p* = 0.048) (Fig. 6c). No significant correlations were found between fALFF and ReHo featured and clinical scales. The results of additional exploratory analyses are reported in the Supplementary Material.

## Discussion

In this study, we explored intrinsic neural activity in SCZ and BD and we examined the association between rs-fMRI parameters and clinical and cognitive features in these individuals. Furthermore, we investigated the potential diagnostic value of fALFF and ReHo in SCZ and BD. We observed a widespread pattern of altered spontaneous brain activity and local connectivity in patients, involving cortical and subcortical structures that were associated with impaired cognition. ReHo performed better than fALFF in distinguishing between the two patient groups and HC, and the stacking model achieved the best performance. The most important features for classifying patients with BD and HC were the ReHo values of the right MFG, right STG, and left anterior insula, and the fALFF values of the right caudate, left transverse temporal gyrus and left thalamus. Differently, for the classification of SCZ and HC, the most important features were the ReHo values of the right hippocampus, right temporal pole, left posterior orbital gyrus, right anterior orbital gyrus, and left cerebellum, as well as the fALFF values of the left ITG, left SMG, right cerebellum, bilateral SPL, and right posterior insula.

### fALFF abnormalities in SCZ and BD

ML analyses showed that the most important features for the classification of SCZ were the fALFF values of the left ITG, left SMG, bilateral SPL, right posterior insula, and right cerebellum. Previous literature showed an increase in fALFF in ITG in patients with SCZ [24], and temporal alterations appear to be associated with hallucinations and delusions in SCZ [39]. Differently, the cerebellum is involved in the motor and cognitive symptoms of SCZ [40]. Interestingly, Schmahmann (1998) and Andreasen et al. (1999) independently suggested a causative role of cerebellum dysfunction for a large number of SCZ symptoms, mediated by impaired coordination of mental and motor processes [41, 42]. In SCZ, altered insular connectivity has been associated with cognitive deficits and negative symptoms [43]. Crucially, our results of altered spontaneous activity in ITG, insula, and cerebellum align with the findings of a recent ML investigation that showed that functional alterations in these areas during a WM task presented a high classification accuracy (> 95%) to distinguish HC and SCZ [44].

Parietal abnormalities appear to play a key role in SCZ, with some studies suggesting that they may appear before prefrontal alterations in individuals with emerging SCZ [45]. In particular, the relevance of SMG in SCZ is not only due to its functions as part of the heteromodal association cortex, but also to its connections to the prefrontal and temporal lobes [46]. Several studies have reported findings of abnormal SPG activity in SCZ, both during performance of a task and during rest [47, 48]. Importantly, a recent study that used structural and functional MRI features to discriminate SCZ and HC showed that the most discriminative regions were the left SPL, the SMG, and the angular gyrus, suggesting that parietal lobe alterations might represent diagnostic markers of SCZ [49].

Regarding BD, the most important features in discriminating between BD and HC were the fALFF values of the right caudate, right precentral gyrus, left thalamus, and left transverse temporal gyrus. It has been shown that caudate displayed increased resting state activity in mania and depression in BD [50, 51], and abnormal caudate FC has also been reported in the first manic episodes [52]. Additionally, changes in the thalamo-striatal network seem to be involved in cognitive deficits in BD [53, 54]. Overall, resting-state alterations in the thalamostriatal network appear to be promising features not only for the discrimination of BD from HC, but also for the identification of specific subsets of patients with BD. Also, albeit exploratory, we showed a correlation between verbal learning and WM and thalamo-striatal fALFF values, underscoring the involvement of this network in high-order cognitive functions.

Lastly, the left transverse temporal gyrus emerged as an area that could discriminate BD from HC. Functional alterations of the transverse temporal gyrus have been reported in BD and are similar to those observed in SCZ [55]. Notably, resting-state changes in the auditory cortex have been associated with hallucination severity across the psychosis spectrum [56]. Overall, fALFF abnormalities in the left transverse temporal gyrus seem to have a discriminatory value in BD, suggesting that the auditory cortex could be involved in the pathophysiology of BD.

Not surprisingly, we found a negative correlation between component 1 and component 2 scores and the fALFF values of the left transverse temporal gyrus, indicating a role for this area in altered memory and verbal learning in BD.

Both SCZ and BD showed higher fALFF in the right precentral gyrus relative to HC. This result is in line with previous studies showing convergent abnormalities in the precentral gyrus in SCZ and BD, indicating shared sensory-motor alterations [57]. Also, exploratory analyses showed a negative correlation in BD between memory, verbal learning, and WM and the fALFF values of the right precentral gyrus, which might suggest that functional alterations in this region could be associated with disorder-specific cognitive alterations.

### ReHo abnormalities in SCZ and BD

For the classification of SCZ and HC, the most important features were the ReHo values of the right hippocampus, left posterior orbital gyrus, right anterior orbital gyrus, right temporal pole, and left cerebellum. Several lines of research suggest that hippocampal abnormalities are involved in the pathophysiology of SCZ [58]. Furthermore, aberrant intra- and internetwork FC patterns of the hippocampus have been observed in SCZ [59] and structural features of the bilateral hippocampal subfields have been shown to accurately differentiate patients with SCZ from HC [60, 61]. Similarly, resting-state alterations in the orbitofrontal gyrus have been commonly reported in SCZ and appear to be related to deficits in cognition, decision-making, and sensory information processing [62, 63]. In particular, a seed-based FC study that performed feature extraction from rs-fMRI data and applied ML classifiers in individuals with SCZ with and without a history of auditory hallucinations and HC showed that classification was possible with high precision. Importantly, the orbitofrontal gyrus emerged as a highly discriminant in classification experiments. In addition, the temporal regions were highly discriminant, with the left middle temporal gyrus and right temporal pole emerging as significant [64]. In SCZ, temporal pole abnormalities have been associated with the severity of disorganized symptoms, delusions, and hallucinations [65] and with alterations in social and emotional processing [66], suggesting that the temporal pole might present diagnostic potential for SCZ, in particular in those individuals with manifest psychotic symptoms.

Unlike fALFF analyses, which showed differences between SCZ and HC in the right cerebellum, the ReHo differences were located in the left cerebellum. These results suggest that fALFF and ReHo, which are distinct, albeit complementary, measures, could be differentially sensitive to the features of resting brain properties, with fALFF capturing spontaneous neural activity and ReHo measuring the short-range connectivity of the local BOLD signal. Interestingly, a recent investigation that examined the discriminatory role of ReHo for psychosis biotypes validated ReHo as a potential neuroimaging biomarker in biotypes and reported generally stronger separation with ReHo between biotypes and between patients and controls compared to conventional diagnostic systems [67], highlighting the potential of ReHo to serve as a biomarker to distinguish between patients with SCZ and HC.

ML analyses in BD demonstrated that the most important features for classifying BD and HC were the ReHo values of the right MFG, right STG, and left anterior insula. The MFG, a region involved in higher cognitive functions such as attention shifting, WM, and response inhibition [68], which were found to be abnormal in BD patients also during remission [69, 70], displays within-network FC aberrancies in BD [71]. Increases in ReHo in the STG have previously been reported in drug-naïve BD [72]. Additionally, the insula has frequently been implicated in BD, with changes in ReHo in the insula often reported in BD [73, 74]. Notably, the anterior insula is part of the salience network and is also functionally connected to the dorsolateral prefrontal cortex [75]. Interestingly, exploratory analyses showed a negative correlation between component 1 and component 2 scores and the ReHo values right MFG, right STG, and left anterior insula, indicating a role of these changes not only in the development of psychopathology but also in specific cognitive deficits of BD. Overall, in BD, ReHo changes in prefronto-temporal and insular regions seem to present discriminatory power. These results are consistent with a recent ML investigation that showed that a combination of structural and ReHo changes in the prefronto, temporal, striatal, and limbic lobe could effectively identify patients with BD with an accuracy of 87.5%, a sensitivity of 86.4%, and a specificity of 88.9% [76].

A direct comparison of SCZ and BD showed a decrease in ReHo in SCZ compared to BD in the right calcarine scissure, while both SCZ and BD had lower ReHo in the right IOG compared to HC.

The visual system plays a key role in the pathology of psychotic disorders. Several studies have reported impairments at multiple levels of visual processing, ranging from perceptual integration to speed discrimination and higher-order visual functions, which are associated with positive and negative psychotic symptoms [77]. Furthermore, early visual processing deficits have been shown to affect higher-order cognitive functions, including emotion recognition, through bottom-up mechanisms [78]. Interestingly, visual impairments in childhood and adolescence are linked to the development of SCZ later in life [79]. In line with this evidence, we observed that SCZ and BD presented a decrease in ReHo in the IOG, a region structurally connected with the limbic system with a central role in facial emotion processing [80, 81]. Additionally, SCZ specifically presented a reduction in ReHo in the right calcarine scissure, an area implicated in reality monitoring, defined as the process that allows us to distinguish imagination and thoughts from the information we perceive from the environment [82]. This evidence aligns with the core psychopathological features of the two disorders and suggests that both SCZ and BD show changes in brain regions involved in affective processing, while only SCZ shows alterations in brain regions implicated in reality monitoring, which could be associated with delusions and hallucinations.

### Diagnostic value of fALFF and ReHo

Intrinsic brain activity measures showed different abilities to distinguish diagnostic groups. Although ReHo features had better discriminatory power compared to fALFF, the integration of these modalities showed the most robust classification performance among patients [83]. This finding underscores the complementary information encoded by the ReHo and fALFF metrics and highlights the potential for multivariate integration to improve diagnostic accuracy [84]. The ability of the stacking model to leverage the advantages of individual modalities further emphasizes the efficacy of ensemble methods for unraveling intricate neurobiological signatures in complex psychiatric conditions [85]. Interestingly, we found that the ML classifiers discriminating between healthy individuals and patients could not be generalized between BD and SCZ since in all models discriminatory scores differed between diagnoses, thus suggesting that the neuroimaging signatures were specific for each disorder.

Lastly, when differentiating between SCZ and BD using neuroimaging and clinical data, the latter showed better discriminatory performance, with better detection through clinical scales assessing specific domains such as bizarre behaviors, delusions, hallucinations, and depressed mood. This could be due to shared structural and functional similarities [86, 87] and to the specificities in the clinical presentation [88] of the two disorders.

## Limitations and conclusions

The study sample size, the detailed cognitive assessment, and the use of a comprehensive set of sophisticated FC measures represent the strengths of our study. However, some methodological aspects limit the generalizability of our results. First, we cannot exclude an effect of treatment on FC, especially in regions and networks involved in sensorimotor and cognitive processing, which are known to be responsible for the effect of several commonly used medications for these disorders [89, 90]. Second, illness progression could also exert an effect on functional properties [91], along with other factors known to be related to both disorders, such as cigarette smoking [92]. Third, while nested cross-validation with SVM analysis was employed to enhance the result reliability, restricting the analysis to regions exhibiting significant between-group differences introduces some constraints. Future investigations should consider larger sample sizes, incorporate additional features, and employ independent datasets for further validation of the findings.

In conclusion, our findings support altered local and global spontaneous activity in widespread cortical and subcortical brain networks in the pathophysiology of SCZ and BD, with more relevant changes in local connectivity within fronto-temporal networks. Furthermore, they suggest a complementary role for spontaneous activity measures in the classification of these patients and a potential of their multivariate combination to improve diagnostic precision.

## Electronic supplementary material

Below is the link to the electronic supplementary material.

Supplementary Material 1

Supplementary Material 2

## References

[1] Lichtenstein P, Yip BH, Björk C et al (2009) Common genetic determinants of schizophrenia and bipolar disorder in Swedish families: a population-based study. Lancet 373:234–239. 10.1016/S0140-6736(09)60072-6 19150704 10.1016/S0140-6736(09)60072-6 PMC3879718

[2] Murray RM, Sham P, Van Os J et al (2004) A developmental model for similarities and dissimilarities between schizophrenia and bipolar disorder. Schizophr Res 71:405–416. 10.1016/J.SCHRES.2004.03.002 15474912 10.1016/j.schres.2004.03.002

[3] Arnone D, Cavanagh J, Gerber D et al (2009) Magnetic resonance imaging studies in bipolar disorder and schizophrenia: meta-analysis. Br J Psychiatry 195:194–201. 10.1192/BJP.BP.108.059717 19721106 10.1192/bjp.bp.108.059717

[4] Shenton ME, Dickey CC, Frumin M, McCarley RW (2001) A review of MRI findings in schizophrenia. Schizophr Res 49:1–52. 10.1016/S0920-9964(01)00163-3 11343862 10.1016/s0920-9964(01)00163-3 PMC2812015

[5] Fountoulakis KN, Giannakopoulos P, Kövari E, Bouras C (2008) Assessing the role of cingulate cortex in bipolar disorder: neuropathological, structural and functional imaging data. Brain Res Rev 59:9–21. 10.1016/J.BRAINRESREV.2008.04.005 18539335 10.1016/j.brainresrev.2008.04.005

[6] O’Donoghue S, Holleran L, Cannon DM, McDonald C (2017) Anatomical dysconnectivity in bipolar disorder compared with schizophrenia: a selective review of structural network analyses using diffusion MRI. J Affect Disord 209:217–228. 10.1016/J.JAD.2016.11.015 27930915 10.1016/j.jad.2016.11.015

[7] Cattarinussi G, Kubera KM, Hirjak D et al (2022) Neural correlates of the risk for Schizophrenia and bipolar disorder: a meta-analysis of structural and functional neuroimaging studies. Biol Psychiatry. 10.1016/J.BIOPSYCH.2022.02.960 35523593 10.1016/j.biopsych.2022.02.960

[8] Heeger DJ, Ress D (2002) What does fMRI tell us about neuronal activity? Nature Reviews Neuroscience 2002 3:2 3:142–151. 10.1038/nrn730 10.1038/nrn730 11836522

[9] Biswal B, Zerrin Yetkin F, Haughton VM, Hyde JS (1995) Functional connectivity in the motor cortex of resting human brain using echo-planar MRI. Magn Reson Med 34:537–541. 10.1002/MRM.1910340409 8524021 10.1002/mrm.1910340409

[10] Shen HH (2015) Core concept: resting-state connectivity. Proc Natl Acad Sci U S A 112:14115–14116. 10.1073/PNAS.1518785112/ASSET/9CBD400E-A669-4DB3-A57F-D82ACFC9E068/ASSETS/GRAPHIC/PNAS.1518785112FIG01.JPEG 26578753 10.1073/pnas.1518785112 PMC4655520

[11] Fristen KJ, Frith CD, Fletcher P et al (1996) Functional topography: Multidimensional Scaling and Functional Connectivity in the brain. Cereb Cortex 6:156–164. 10.1093/CERCOR/6.2.156 8670646 10.1093/cercor/6.2.156

[12] Karbasforoushan H, Woodward ND (2013) Resting-state networks in Schizophrenia. Curr Top Med Chem 12:2404–2414. 10.2174/1568026611212210011 10.2174/156802612805289863 23279179

[13] Manoliu A, Riedl V, Zherdin A et al (2014) Aberrant Dependence of Default Mode/Central Executive Network Interactions on Anterior Insular Salience Network Activity in Schizophrenia. Schizophr Bull 40:428–437. 10.1093/schbul/sbt037 23519021 10.1093/schbul/sbt037 PMC3932085

[14] Tang Y, Wang L, Cao F, Tan L (2012) Identify schizophrenia using resting-state functional connectivity: an exploratory research and analysis. Biomed Eng Online 11:1–16. 10.1186/1475-925X-11-50/FIGURES/5 22898249 10.1186/1475-925X-11-50 PMC3462724

[15] Wang Y, Tang W, Fan X et al (2017) Resting-state functional connectivity changes within the default mode network and the salience network after antipsychotic treatment in early-phase schizophrenia. Neuropsychiatr Dis Treat 13:397–406. 10.2147/NDT.S123598 28223812 10.2147/NDT.S123598 PMC5308583

[16] Syan SK, Smith M, Frey BN et al (2018) Resting-state functional connectivity in individuals with bipolar disorder during clinical remission: a systematic review. J Psychiatry Neurosci 43:298. 10.1503/JPN.170175 30125243 10.1503/jpn.170175 PMC6158027

[17] Di X, Kim EH, Huang CC et al (2013) The influence of the amplitude of low-frequency fluctuations on resting-state functional connectivity. Front Hum Neurosci 7:42610. 10.3389/FNHUM.2013.00118/BIBTEX 10.3389/fnhum.2013.00118 PMC3613753 23565090

[18] Zou QH, Zhu CZ, Yang Y et al (2008) An improved approach to detection of amplitude of low-frequency fluctuation (ALFF) for resting-state fMRI: fractional ALFF. J Neurosci Methods 172:137–141. 10.1016/J.JNEUMETH.2008.04.012 18501969 10.1016/j.jneumeth.2008.04.012 PMC3902859

[19] Zuo XN, Di Martino A, Kelly C et al (2010) The oscillating brain: Complex and reliable. NeuroImage 49:1432–1445. 10.1016/J.NEUROIMAGE.2009.09.037 19782143 10.1016/j.neuroimage.2009.09.037 PMC2856476

[20] Zang Y, Jiang T, Lu Y et al (2004) Regional homogeneity approach to fMRI data analysis. NeuroImage 22:394–400. 10.1016/j.neuroimage.2003.12.030 15110032 10.1016/j.neuroimage.2003.12.030

[21] Deng S, Franklin CG, O’Boyle M et al (2022) Hemodynamic and metabolic correspondence of resting-state voxel-based physiological metrics in healthy adults. NeuroImage 250:118923. 10.1016/j.neuroimage.2022.118923 35066157 10.1016/j.neuroimage.2022.118923 PMC9201851

[22] Xie J, Zhang W, Shen Y et al (2023) Abnormal spontaneous brain activity in females with autism spectrum disorders. Front Neurosci 17:1189087. 10.3389/FNINS.2023.1189087/BIBTEX 37521682 10.3389/fnins.2023.1189087 PMC10379634

[23] Liu C, Xue Z, Palaniyappan L et al (2016) Abnormally increased and incoherent resting-state activity is shared between patients with schizophrenia and their unaffected siblings. Schizophr Res 171:158–165. 10.1016/J.SCHRES.2016.01.022 26805410 10.1016/j.schres.2016.01.022

[24] Xu Y, Zhuo C, Qin W et al (2015) Altered spontaneous brain activity in Schizophrenia: a Meta-analysis and a large-sample study. Biomed Res Int 2015. 10.1155/2015/204628 10.1155/2015/204628 PMC4477065 26180786

[25] Cai M, Wang R, Liu M et al (2022) Disrupted local functional connectivity in schizophrenia: an updated and extended meta-analysis. Schizophrenia (Heidelberg Germany) 8. 10.1038/S41537-022-00311-2 10.1038/s41537-022-00311-2 PMC9643538 36347874

[26] Hui Y, Jize Xiang Hui Yang zHenggui zHou Yu Wu Yong zHang, Maolan J (2021) Explore functional brain changes in bipolar disorder: A whole brain ALE meta-analysis. Archives of Clinical Psychiatry 48:208–215. 10.15761/0101-60830000000309

[27] Dwyer DB, Falkai P, Koutsouleris N (2018) Machine learning approaches for clinical psychology and Psychiatry. Annu Rev Clin Psychol 14:91–118. 10.1146/ANNUREV-CLINPSY-032816-045037 29401044 10.1146/annurev-clinpsy-032816-045037

[28] Mechelli Andrea V (2020) Sandra Machine learning: methods and applications to brain disorders

[29] Sabaroedin K, Razi A, Chopra S et al (2023) Frontostriatothalamic effective connectivity and dopaminergic function in the psychosis continuum. Brain 146:372–386. 10.1093/BRAIN/AWAC018 35094052 10.1093/brain/awac018 PMC9825436

[30] Hamilton M (1960) A rating scale for depression. J Neurol Neurosurg Psychiatry 23:56–62. 10.1136/JNNP.23.1.56 14399272 10.1136/jnnp.23.1.56 PMC495331

[31] Andersen NC (1989) The Scale for the Assessment of negative symptoms (SANS): conceptual and theoretical foundations. Br J Psychiatry Suppl 49–58 2695141

[32] Andersen N (1984) The Scale for the Assessment of positive symptoms. SAPS)

[33] Young RC, Biggs JT, Ziegler VE, Meyer DA (1978) A rating scale for mania: reliability, validity and sensitivity. Br J Psychiatry 133:429–435. 10.1192/BJP.133.5.429 728692 10.1192/bjp.133.5.429

[34] Zang YF, Yong H, Chao-Zhe Z et al (2007) Altered baseline brain activity in children with ADHD revealed by resting-state functional MRI. Brain Dev 29:83–91. 10.1016/J.BRAINDEV.2006.07.002 16919409 10.1016/j.braindev.2006.07.002

[35] Cabral C, Kambeitz-Ilankovic L, Kambeitz J et al (2016) Classifying Schizophrenia using Multimodal Multivariate Pattern Recognition Analysis: evaluating the impact of individual clinical profiles on the Neurodiagnostic performance. Schizophr Bull 42:S110–S117. 10.1093/SCHBUL/SBW053 27460614 10.1093/schbul/sbw053 PMC4960438

[36] Koutsouleris N, Riecher-Rössler A, Meisenzahl EM et al (2015) Detecting the psychosis Prodrome Across High-Risk populations using neuroanatomical biomarkers. Schizophr Bull 41:471. 10.1093/SCHBUL/SBU078 24914177 10.1093/schbul/sbu078 PMC4332937

[37] Raio A, Pergola G, Rampino A et al (2023) Similarities and differences between multivariate patterns of cognitive and socio-cognitive deficits in schizophrenia, bipolar disorder and related risk. Schizophrenia 2023 9(1):1–13. 10.1038/s41537-023-00337-0 10.1038/s41537-023-00337-0 PMC9938280 36801866

[38] De Filippis R, Carbone EA, Gaetano R et al (2019) Machine learning techniques in a structural and functional MRI diagnostic approach in schizophrenia: a systematic review. Neuropsychiatr Dis Treat 15:1605–1627. 10.2147/NDT.S202418 31354276 10.2147/NDT.S202418 PMC6590624

[39] Sun J, Maller JJ, Guo L, Fitzgerald PB (2009) Superior temporal gyrus volume change in schizophrenia: a review on region of interest volumetric studies. Brain Res Rev 61:14–32. 10.1016/J.BRAINRESREV.2009.03.004 19348859 10.1016/j.brainresrev.2009.03.004

[40] Shinn AK, Baker JT, Lewandowski KE et al (2015) Aberrant cerebellar connectivity in motor and association networks in schizophrenia. Front Hum Neurosci 9:121129. 10.3389/FNHUM.2015.00134/ABSTRACT 10.3389/fnhum.2015.00134 PMC4364170 25852520

[41] Andreasen NC, Nopoulos P, O’Leary DS et al (1999) Defining the phenotype of schizophrenia: cognitive dysmetria and its neural mechanisms. Biol Psychiatry 46:908–920. 10.1016/S0006-3223(99)00152-3 10509174 10.1016/s0006-3223(99)00152-3

[42] Schmahmann JD (1998) Dysmetria of thought: clinical consequences of cerebellar dysfunction on cognition and affect. Trends Cogn Sci 2:362–371. 10.1016/S1364-6613(98)01218-2 21227233 10.1016/s1364-6613(98)01218-2

[43] Sheffield JM, Rogers BP, Blackford JU et al (2020) Insula Functional Connectivity in Schizophrenia. Schizophr Res 220:69. 10.1016/J.SCHRES.2020.03.068 32307263 10.1016/j.schres.2020.03.068 PMC7322763

[44] Chatterjee I, Kumar V, Sharma S et al (2019) Identification of brain regions associated with working memory deficit in schizophrenia. 10.12688/F1000RESEARCH.17731.1/DOI. F1000Res 8:10.12688/f1000research.17731.1 PMC6480944 31069066

[45] Yildiz M, Borgwardt SJ, Berger GE (2011) Parietal lobes in Schizophrenia: do they matter? Schizophr Res Treat 2011:1–15. 10.1155/2011/581686 10.1155/2011/581686 PMC3420742 22937268

[46] Rolls ET, Deco G, Huang C-C, Feng J (2023) The human posterior parietal cortex: effective connectome, and its relation to function. Cereb Cortex 33:3142–3170. 10.1093/CERCOR/BHAC266 35834902 10.1093/cercor/bhac266 PMC10401905

[47] Holt DJ, Boeke EA, Coombs G et al (2015) Abnormalities in personal space and parietal–frontal function in schizophrenia. Neuroimage Clin 9:233–243. 10.1016/J.NICL.2015.07.008 26484048 10.1016/j.nicl.2015.07.008 PMC4573090

[48] Ojeda N, Ortuño F, Arbizu J et al (2002) Functional neuroanatomy of sustained attention in schizophrenia: contribution of parietal cortices. Hum Brain Mapp 17:116. 10.1002/HBM.10055 12353245 10.1002/hbm.10055 PMC6871970

[49] Shi D, Li Y, Zhang H et al (2021) Machine Learning of Schizophrenia Detection with structural and functional neuroimaging. 10.1155/2021/9963824. Dis Markers 2021:10.1155/2021/9963824 PMC8208855 34211615

[50] Blumberg HP, Stem E, Martinez D et al (2019) Increased anterior cingulate and caudate activity in bipolar mania. Bipolar Disorder: The Science of Mental Health 177–184. 10.4324/9781315054308-17/INCREASED-ANTERIOR-CINGULATE-CAUDATE-ACTIVITY-BIPOLAR-MANIA-HILARY-BLUMBERG-EMILY-STEM-DIANA-MARTINEZ-SALLY-RICKETTS-JOSE-DE-ASIS-THOMAS-WHITE-JANE-EPSTEIN-ANNE-MCBRIDE-DAVID-EIDELBERG-JAMES-KOCSIS-DAVID-SILBERSWEIG

[51] He Z, W S, F L, et al (2019) Altered resting-state cerebral blood flow and functional connectivity of striatum in bipolar disorder and major depressive disorder. Prog Neuropsychopharmacol Biol Psychiatry 90:177–185. 10.1016/J.PNPBP.2018.11.009 30500413 10.1016/j.pnpbp.2018.11.009

[52] Lippard ETC, Weber W, Welge J et al (2021) Variation in rostral anterior cingulate functional connectivity with amygdala and caudate during first manic episode distinguish bipolar young adults who do not remit following treatment. Bipolar Disord 23:500–508. 10.1111/BDI.13025 33089593 10.1111/bdi.13025 PMC8060357

[53] Haznedar MM, Roversi F, Pallanti S et al (2005) Fronto-thalamo-striatal gray and white matter volumes and anisotropy of their connections in bipolar spectrum illnesses. Biol Psychiatry 57:733–742. 10.1016/J.BIOPSYCH.2005.01.002 15820230 10.1016/j.biopsych.2005.01.002

[54] Teng S, Lu CF, Wang PS et al (2014) Altered Resting-State Functional Connectivity of Striatal-Thalamic Circuit in Bipolar Disorder. PLoS ONE 9:e96422. 10.1371/JOURNAL.PONE.0096422 24788849 10.1371/journal.pone.0096422 PMC4008631

[55] Reite M, Teale P, Rojas DC et al (2009) MEG auditory evoked fields suggest altered structural/functional asymmetry in primary but not secondary auditory cortex in bipolar disorder. Bipolar Disord 11:371–381. 10.1111/J.1399-5618.2009.00701.X 19500090 10.1111/j.1399-5618.2009.00701.x PMC2905653

[56] Okuneye VT, Meda S, Pearlson GD et al (2020) Resting state auditory-language cortex connectivity is associated with hallucinations in clinical and biological subtypes of psychotic disorders. Neuroimage Clin 27. 10.1016/J.NICL.2020.102358 10.1016/j.nicl.2020.102358 PMC7398970 32745995

[57] Jørgensen KN, Nerland S, Norbom LB et al (2016) Increased MRI-based cortical grey/white-matter contrast in sensory and motor regions in schizophrenia and bipolar disorder. Psychol Med 46:1971–1985. 10.1017/S0033291716000593 27049014 10.1017/S0033291716000593

[58] Lieberman JA, Girgis RR, Brucato G et al (2018) Hippocampal dysfunction in the pathophysiology of schizophrenia: a selective review and hypothesis for early detection and intervention. Mol Psychiatry 2018 23:8. 10.1038/mp.2017.249 10.1038/mp.2017.249 PMC6037569 29311665

[59] Li XW, Liu H, Deng YY et al (2023) Aberrant intra- and internetwork functional connectivity patterns of the anterior and posterior hippocampal networks in schizophrenia. CNS Neurosci Ther 29:2223. 10.1111/CNS.14171 36949599 10.1111/cns.14171 PMC10352891

[60] Nieuwenhuis M, van Haren NEM, Hulshoff Pol HE et al (2012) Classification of schizophrenia patients and healthy controls from structural MRI scans in two large independent samples. NeuroImage 61:606–612. 10.1016/J.NEUROIMAGE.2012.03.079 22507227 10.1016/j.neuroimage.2012.03.079

[61] Park YW, Choi D, Lee J et al (2020) Differentiating patients with schizophrenia from healthy controls by hippocampal subfields using radiomics. Schizophr Res 223:337–344. 10.1016/J.SCHRES.2020.09.009 32988740 10.1016/j.schres.2020.09.009

[62] Chen C, Yao J, Lv Y et al (2022) Aberrant functional connectivity of the Orbitofrontal cortex is Associated with excited symptoms in first-episode Drug-Naïve patients with Schizophrenia. Front Psychiatry 13. 10.3389/FPSYT.2022.922272 10.3389/fpsyt.2022.922272 PMC9366470 35966466

[63] Giordano GM, Pezzella P, Giuliani L et al (2023) Resting-state brain activity dysfunctions in Schizophrenia and their associations with negative Symptom domains: an fMRI study. Brain Sci 13. 10.3390/BRAINSCI13010083 10.3390/brainsci13010083 PMC9856573 36672064

[64] Chyzhyk D, Graña M, Öngür D, Shinn AK (2015) Discrimination of schizophrenia auditory hallucinators by machine learning of resting-state functional MRI. Int J Neural Syst 25. 10.1142/S0129065715500070 10.1142/S0129065715500070 PMC4787625 25753600

[65] Crespo-Facorro B, Nopoulos PC, Chemerinski E et al (2004) Temporal Pole morphology and psychopathology in males with schizophrenia. Psychiatry Res 132:107–115. 10.1016/J.PSCYCHRESNS.2004.09.002 15598545 10.1016/j.pscychresns.2004.09.002

[66] Guo P, Hu S, Jiang X et al (2022) Associations of Neurocognition and Social Cognition with Brain structure and function in early-onset Schizophrenia. Front Psychiatry 13:798105. 10.3389/FPSYT.2022.798105/FULL 35222115 10.3389/fpsyt.2022.798105 PMC8866448

[67] Ji L, Meda SA, Tamminga CA et al (2020) Characterizing functional regional homogeneity (ReHo) as a B-SNIP psychosis biomarker using traditional and machine learning approaches. Schizophr Res 215:430–438. 10.1016/J.SCHRES.2019.07.015 31439419 10.1016/j.schres.2019.07.015

[68] Kobayashi S (2009) Reward neurophysiology and primate cerebral cortex. Encyclopedia Neurosci 325–333. 10.1016/B978-008045046-9.01559-X

[69] Hidiroğlu C, Torres IJ, Er A et al (2015) Response inhibition and interference control in patients with bipolar I disorder and first-degree relatives. Bipolar Disord 17:781–794. 10.1111/BDI.12335 26415581 10.1111/bdi.12335

[70] Soraggi-Frez C, Santos FH, Albuquerque PB, Malloy-Diniz LF (2017) Disentangling working memory functioning in mood states of bipolar disorder: a systematic review. Front Psychol 8:228016. 10.3389/FPSYG.2017.00574/BIBTEX 10.3389/fpsyg.2017.00574 PMC5405335 28491042

[71] Gong J, Wang J, Chen P et al (2021) Large-scale network abnormality in bipolar disorder: a multimodal meta-analysis of resting-state functional and structural magnetic resonance imaging studies. J Affect Disord 292:9–20. 10.1016/J.JAD.2021.05.052 34087634 10.1016/j.jad.2021.05.052

[72] Shan X, Qiu Y, Pan P et al (2020) Disrupted Regional Homogeneity in Drug-naive patients with bipolar disorder. 10.3389/FPSYT.2020.00825. Front Psychiatry 11:10.3389/fpsyt.2020.00825 PMC7456987 32922322

[73] Achalia RM, Jacob A, Achalia G et al (2019) Investigating spontaneous brain activity in bipolar disorder: a resting-state functional magnetic resonance imaging study. Indian J Psychiatry 61:630–634. 10.4103/PSYCHIATRY.INDIANJPSYCHIATRY_391_19 31896871 10.4103/psychiatry.IndianJPsychiatry_391_19 PMC6862975

[74] Xiao Q, Zhong Y, Lu D et al (2013) Altered Regional Homogeneity in Pediatric Bipolar disorder during Manic State: a resting-state fMRI study. PLoS ONE 8:1–9. 10.1371/journal.pone.0057978 10.1371/journal.pone.0057978 PMC3590243 23526961

[75] Pastrnak M, Simkova E, Novak T (2021) Insula activity in resting-state differentiates bipolar from unipolar depression: a systematic review and meta-analysis. Scientific Reports 2021 11:1 11:1–11. 10.1038/s41598-021-96319-2 10.1038/s41598-021-96319-2 PMC8379217 34417487

[76] Li H, Cui L, Cao L et al (2020) Identification of bipolar disorder using a combination of multimodality magnetic resonance imaging and machine learning techniques. BMC Psychiatry 20. 10.1186/S12888-020-02886-5 10.1186/s12888-020-02886-5 PMC7542439 33023515

[77] Türközer HB, Lizano P, Adhan I et al (2022) Regional and Sex-specific alterations in the visual cortex of individuals with psychosis Spectrum disorders. Biol Psychiatry 92:396–406. 10.1016/J.BIOPSYCH.2022.03.023 35688762 10.1016/j.biopsych.2022.03.023

[78] Butler PD, Abeles IY, Weiskopf NG et al (2009) Sensory contributions to impaired emotion Processing in Schizophrenia. Schizophr Bull 35:1095–1107. 10.1093/SCHBUL/SBP109 19793797 10.1093/schbul/sbp109 PMC2762631

[79] Hayes JF, Picot S, Osborn DPJ et al (2019) Visual acuity in late adolescence and future psychosis risk in a cohort of 1 million men. Schizophr Bull 45:571–578. 10.1093/schbul/sby084 29901774 10.1093/schbul/sby084 PMC6483575

[80] Sato W, Kochiyama T, Uono S et al (2017) Bidirectional electric communication between the inferior occipital gyrus and the amygdala during face processing. Hum Brain Mapp 38:4511. 10.1002/HBM.23678 28573679 10.1002/hbm.23678 PMC6867177

[81] Xiu D, Geiger MJ, Kiaver P (2015) Emotional face expression modulates occipital-frontal effective connectivity during memory formation in a bottom-up fashion. Front Behav Neurosci 9:127130. 10.3389/FNBEH.2015.00090/ABSTRACT 10.3389/fnbeh.2015.00090 PMC4407577 25954169

[82] Lavallé L, Brunelin J, Jardri R et al (2023) The neural signature of reality-monitoring: a meta‐analysis of functional neuroimaging studies. Hum Brain Mapp 44:4372. 10.1002/HBM.26387 37246722 10.1002/hbm.26387 PMC10318245

[83] Pavlyshenko BM (2019) Machine-Learning Models for Sales Time Series Forecasting. Data 2019, Vol 4, Page 15 4:15. 10.3390/DATA4010015

[84] Golestani AM, Kwinta JB, Khatamian YB, Chen JJ (2017) The effect of low-frequency physiological correction on the reproducibility and specificity of resting-state fMRI Metrics: functional connectivity, ALFF, and ReHo. 10.3389/FNINS.2017.00546. Front Neurosci 11:10.3389/fnins.2017.00546 PMC5633680 29051724

[85] Rokach L (2010) Ensemble-based classifiers. Artif Intell Rev 33:1–39. 10.1007/S10462-009-9124-7/METRICS

[86] Argyelan M, Ikuta T, Derosse P et al (2014) Resting-state fMRI connectivity impairment in Schizophrenia and Bipolar Disorder. Schizophr Bull 40:100–110. 10.1093/SCHBUL/SBT092 23851068 10.1093/schbul/sbt092 PMC3885301

[87] Rootes-Murdy K, Edmond JT, Jiang W et al (2022) Clinical and cortical similarities identified between bipolar disorder I and schizophrenia: a multivariate approach. Front Hum Neurosci 16. 10.3389/FNHUM.2022.1001692/FULL 10.3389/fnhum.2022.1001692 PMC9684186 36438633

[88] American Psychiatric Association (2022) Diagnostic and Statistical Manual of Mental Disorders. 10.1176/APPI.BOOKS.9780890425787

[89] Baizabal-Carvallo JF, Alonso-Juarez M (2021) Valproate-induced rest tremor and parkinsonism. Acta Neurol Belg 121:515–519. 10.1007/S13760-019-01239-8/TABLES/3 31721077 10.1007/s13760-019-01239-8

[90] Baldez DP, Biazus TB, Rabelo-da-Ponte FD et al (2021) The effect of antipsychotics on the cognitive performance of individuals with psychotic disorders: Network meta-analyses of randomized controlled trials. Neurosci Biobehav Rev 126:265–275. 10.1016/J.NEUBIOREV.2021.03.028 33812977 10.1016/j.neubiorev.2021.03.028

[91] Li T, Wang Q, Zhang J et al (2017) Brain-wide analysis of functional connectivity in First-Episode and chronic stages of Schizophrenia. Schizophr Bull 43:436–448. 10.1093/SCHBUL/SBW099 27445261 10.1093/schbul/sbw099 PMC5605268

[92] Chen J, Chen R, Xiang S et al (2021) Cigarette smoking and schizophrenia: mendelian randomisation study. Br J Psychiatry 218:98–103. 10.1192/BJP.2020.116 32552923 10.1192/bjp.2020.116

