# Supplementary material for: Diagnostic value of regional homogeneity and fractional amplitude of low-frequency fluctuations in the classification of schizophrenia and bipolar disorders
Source: Eur Arch Psychiatry Clin Neurosci. 2024 Jun 25;275(3):799–812. doi: 10.1007/s00406-024-01838-4 (PMC11947052; doi:10.1007/s00406-024-01838-4)

**Diagnostic Value of Regional Homogeneity and Fractional Amplitude of Low-Frequency Fluctuations in the Classification of Schizophrenia and Bipolar Disorders**

*Supplementary Material*

Contents:

Supplementary Results

Supplementary Tables S.1-S.6

Supplementary Figures S.1-S.4

**Supplementary Results**

*Association between abnormal fALFF and ReHo values and clinical symptoms and cognitive deficits in SCZ and BD*

An exploratory analysis was conducted to explore the correlations between fALFF and ReHo values and clinical and cognitive scores. In SCZ, the ReHo values of the left temporal pole were negatively correlated with component 1 (rho=-0.350 p=0.037), fALFF values of the left cuneus were positively correlated with component 2 (r=0.35 p=0.02) and fALFF and ReHo values of the right postcentral gyrus were positively correlated with the SAPS scores (rho=0.328 p=0.039; rho=0.322 p=0.043, respectively) (Fig. S.1). In BD, component 1 was negatively correlated with the fALFF values of the left transverse temporal gyrus (rho=-0.316 p=0.04), right caudate (rho=-0.336 p=0.028), and the right precentral gyrus (rho=-0.411 p=0.007), as well as with the ReHo values of the right anterior insula (rho=-0.399 p=0.008), right MFG (rho=-0.390 p=0.01), and the right STG (rho=-0.364 p=0.016) (Fig. S.2). Component 2 was negatively correlated with the fALFF values of the left MFG (rho=-0.331 p=0.031), left thalamus (rho=-0.312 p=0.042), left transverse temporal gyrus (rho=-0.361 p=0.018), right caudate (rho=-0.359 p=0.018), right MFG (rho=-0.315 p=0.040), right precentral gyrus (rho=-0.421 p=0.005), and the ReHo values of the right anterior insula (rho=-0.407 p=0.007), right MFG (rho=-0.355 p=0.019), and the right STG (rho=-0.379 p=0.012) (Fig. S.3). Component 3 was positively correlated with the ReHo values of the right STG (rho=-0.303 p=0.048) (Fig. S.4)

**Table S.1. Clinical characteristics of the sample**

|  | **SCZ**  **n=40** | **BD**  **n=43** | **statistics** | **p** |
| --- | --- | --- | --- | --- |
| HAM-D item 8 (retardation) m (SD) | 0.5 (0.8) | 0.3 (0.6) | U=780 | 0.360 |
| HAM-D item 9  (agitation) m (SD) | 0.9 (1.0) | 0.9 (0.9) | U=832 | 0.788 |
| HAM-D > 8 n (%) | 16 (40%) | 25 (58.1%) | χ2=2.73 | 0.099 |
| YMRS ≥ 20 n (%) | 3 (7.5%) | 12 (27.9%) | χ2=5.83 | 0.016 |

BD: bipolar disorder; HAM-D: Hamilton Depression Scale; m: mean; SCZ: schizophrenia; SD: standard deviation; YMRS: Young Mania Rating Scale.

**Table S.2. Principal component analysis on cognitive tests**

|  | **Component** | | |  |
| --- | --- | --- | --- | --- |
| **Cognitive tests** | **1** | **2** | **3** | **Uniqueness** |
| Wechsler Memory Scale Digit Span total raw score | 1.017 |  |  | 0.0301 |
| Wechsler Memory Digit Span Forward total raw score | 0.959 |  |  | 0.2886 |
| Wechsler Memory Digit Span Longest digit span forward | 0.931 |  |  | 0.339 |
| Wechsler Memory Digit Span Backward total raw score | 0.857 |  |  | 0.293 |
| Wechsler Memory Digit Span Longest digit span backward | 0.855 |  |  | 0.3294 |
| Wechsler Memory Digit Span Sequencing total raw score | 0.722 |  |  | 0.3355 |
| Wechsler Memory Digit Span Longest digit span sequencing | 0.691 |  |  | 0.4101 |
| Verbal working memory max capacity | 0.42 |  |  | 0.6315 |
| Verbal working memory average corrected | 0.375 |  |  | 0.4891 |
| California Verbal Learning Test short delay free recall |  | 0.331 |  | 0.2934 |
| California Verbal Learning Test long delay free recall |  | 0.91 |  | 0.3743 |
| Wechsler Memory Visual Reproduction 2 delayed recall total raw score |  | 0.904 |  | 0.2792 |
| California Verbal Learning Test total corrected |  | 0.9 |  | 0.3924 |
| Wechsler Memory Visual Reproduction 1 immediate recall total raw score |  | 0.862 |  | 0.3015 |
| Wechsler Memory Symbol Span total raw score |  | 0.821 |  | 0.5357 |
| Spatial Working Memory average corrected |  | 0.567 |  | 0.4182 |
| Spatial Working Memory max capacity |  | 0.556 |  | 0.6502 |
| Stop Signal Session Stop*-*signal reaction-time quantity |  |  | 0.95 | 0.1685 |
| Stop Signal Session Stop*-*signal reaction-time |  |  | 0.943 | 0.1738 |
| Continuous Performance Test hits |  |  | -0.398 | 0.8427 |
| Stroop Conflict accuracy effect |  |  | -0.332 | 0.8896 |

**Table S.3. Cognitive performance in SCZ, BD, and HC**

|  |  | **BD** | **HC** | **SCZ** |
| --- | --- | --- | --- | --- |
| **Component 1** |  |  |  |  |
| **BD** | Mean difference | — | -3.37 | 6.81 |
|  | p-value | — | 0.024 | < 0.001 |
| **HC** | Mean difference |  | — | 10.18 |
|  | p-value |  | — | < 0.001 |
| **SCZ** | Mean difference |  |  | — |
|  | p-value |  |  | — |
| **Component 2** |  |  |  |  |
| **BD** | Mean difference | — | -3.74 | 6.26 |
|  | p-value | — | 0.005 | < 0.001 |
| **HC** | Mean difference |  | — | 10.00 |
|  | p-value |  | — | < 0.001 |
| **SCZ** | Mean difference |  |  | — |
|  | p-value |  |  | — |
| **Component 3** |  |  |  |  |
| **BD** | Mean difference | — | 1.50 | -3.74 |
|  | p-value | — | 0.046 | < 0.001 |
| **HC** | Mean difference |  | — | -5.24 |
|  | p-value |  | — | < 0.001 |
| **SCZ** | Mean difference |  |  | — |
|  | p-value |  |  | — |

BD: bipolar disorder; HC: healthy controls; m: mean; SCZ: schizophrenia.

**Table S.4. Correlations between fALFF and ReHo values and chlorpromazine equivalents**

| **fALFF and ReHo values** | **Statistics** | **Equivalents CPZ** |
| --- | --- | --- |
| **fALFF values** | | |
| *SCZ vs. HC* | | |
| left cuneus | Rho Spearman | -0.329 |
|  | p-value | 0.002 |
| left IOG | Rho Spearman | -0.26 |
|  | p-value | 0.018 |
| left MFG | Rho Spearman | -0.112 |
|  | p-value | 0.315 |
| left MFG | Rho Spearman | -0.106 |
|  | p-value | 0.339 |
| left postcentral gyrus | Rho Spearman | -0.127 |
|  | p-value | 0.252 |
| left SMG | Rho Spearman | -0.138 |
|  | p-value | 0.214 |
| left SPL | Rho Spearman | -0.129 |
|  | p-value | 0.245 |
| right precentral gyrus | Rho Spearman | -0.193 |
|  | p-value | 0.081 |
| right SPL | Rho Spearman | -0.059 |
|  | p-value | 0.598 |
| right postcentral gyrus | Rho Spearman | -0.349 |
|  | p-value | 0.001 |
| left caudate | Rho Spearman | 0.161 |
|  | p-value | 0.147 |
| left ITG | Rho Spearman | 0.161 |
|  | p-value | 0.146 |
| left ITG | Rho Spearman | 0.237 |
|  | p-value | 0.031 |
| right cerebellum | Rho Spearman | 0.209 |
|  | p-value | 0.058 |
| right PCC | Rho Spearman | 0.143 |
|  | p-value | 0.198 |
| right posterior insula | Rho Spearman | 0.206 |
|  | p-value | 0.062 |
| *BD vs. HC* | | |
| right IOG | Rho Spearman | 0.078 |
|  | p-value | 0.482 |
| right middle postcentral gyrus | Rho Spearman | 0 |
|  | p-value | 0.999 |
| right occipital fusiform gyrus | Rho Spearman | -0.028 |
|  | p-value | 0.799 |
| left MFG | Rho Spearman | 0.1 |
|  | p-value | 0.368 |
| left thalamus | Rho Spearman | 0.1 |
|  | p-value | 0.367 |
| left transverse gyrus | Rho Spearman | 0.029 |
|  | p-value | 0.794 |
| right caudate | Rho Spearman | 0.07 |
|  | p-value | 0.532 |
| right MFG | Rho Spearman | 0.136 |
|  | p-value | 0.221 |
| right precentral gyrus | Rho Spearman | 0.133 |
|  | p-value | 0.23 |
| *SCZ and BD vs. HC* | | |
| right precentral gyrus | Rho Spearman | 0.253 |
|  | p-value | 0.08 |
| **ReHo values** | | |
| *SCZ vs. HC* | | |
| left SOG | Rho Spearman | -0.227 |
|  | p-value | 0.039 |
| right IOG | Rho Spearman | -0.301 |
|  | p-value | 0.006 |
| right postcentral gyrus | Rho Spearman | -0.209 |
|  | p-value | 0.058 |
| right postcentral gyrus | Rho Spearman | -0.38 |
|  | p-value | < .001 |
| left cerebellum | Rho Spearman | 0.282 |
|  | p-value | 0.01 |
| left posterior orbital gyrus | Rho Spearman | 0.152 |
|  | p-value | 0.171 |
| left temporal pole | Rho Spearman | 0.114 |
|  | p-value | 0.306 |
| right anterior orbital gyrus | Rho Spearman | 0.248 |
|  | p-value | 0.024 |
| right hippocampus | Rho Spearman | 0.242 |
|  | p-value | 0.028 |
| right temporal pole | Rho Spearman | 0.011 |
|  | p-value | 0.923 |
| *BD vs. HC* | | |
| left PCC | Rho Spearman | 0.244 |
|  | p-value | 0.026 |
| right MTG | Rho Spearman | -0.08 |
|  | p-value | 0.47 |
| left anterior insula | Rho Spearman | -0.025 |
|  | p-value | 0.825 |
| right MFG | Rho Spearman | 0.005 |
|  | p-value | 0.965 |
| right STG | Rho Spearman | -0.032 |
|  | p-value | 0.777 |
| *SCZ vs. BD* | | |
| right calcarine scissure | Rho Spearman | -0.26 |
|  | p-value | 0.018 |
| *SCZ and BD vs. HC* | | |
| right IOG | Rho Spearman | 0.366 |
|  | p-value | 0.23 |

BD: bipolar disorder; CPZ: chlorpromazine; HC: healthy controls; IFG: inferior frontal gyrus; IOG: inferior occipital gyrus; ITG: inferior temporal gyrus; MFG: middle frontal gyrus; MTG middle temporal gyrus; PCC: posterior cingulate cortex; SCZ: schizophrenia; SMG: supramarginal gyrus; SOG: superior occipital gyrus; SPL: superior parietal gyrus.

**Table S.5. Correlations between fALFF and ReHo values and mood stabilizers DDD**

| **fALFF and ReHo values** | **Statistics** | **Mood stabilizers DDD** |
| --- | --- | --- |
| **fALFF values** | | |
| *SCZ vs. HC* | | |
| left cuneus | Rho Spearman | 0.097 |
|  | p-value | 0.381 |
| left IOG | Rho Spearman | 0.115 |
|  | p-value | 0.3 |
| left MFG | Rho Spearman | -0.117 |
|  | p-value | 0.292 |
| left MFG | Rho Spearman | 0.116 |
|  | p-value | 0.295 |
| left postcentral gyrus | Rho Spearman | 0.133 |
|  | p-value | 0.23 |
| left SMG | Rho Spearman | 0.043 |
|  | p-value | 0.697 |
| left SPL | Rho Spearman | 0.028 |
|  | p-value | 0.804 |
| right precentral gyrus | Rho Spearman | -0.045 |
|  | p-value | 0.684 |
| right SPL | Rho Spearman | -0.043 |
|  | p-value | 0.701 |
| right postcentral gyrus | Rho Spearman | 0.032 |
|  | p-value | 0.776 |
| left caudate | Rho Spearman | 0.069 |
|  | p-value | 0.533 |
| left ITG | Rho Spearman | 0.135 |
|  | p-value | 0.223 |
| left ITG | Rho Spearman | 0.201 |
|  | p-value | 0.069 |
| right cerebellum | Rho Spearman | 0.05 |
|  | p-value | 0.653 |
| right PCC | Rho Spearman | 0.01 |
|  | p-value | 0.929 |
| right posterior insula | Rho Spearman | 0.09 |
|  | p-value | 0.419 |
| *BD vs. HC* | | |
| right IOG | Rho Spearman | -0.169 |
|  | p-value | 0.127 |
| right middle postcentral gyrus | Rho Spearman | -0.194 |
|  | p-value | 0.078 |
| right occipital fusiform gyrus | Rho Spearman | 0.028 |
|  | p-value | 0.804 |
| left MFG | Rho Spearman | 0.157 |
|  | p-value | 0.157 |
| left thalamus | Rho Spearman | 0.084 |
|  | p-value | 0.449 |
| left transverse gyrus | Rho Spearman | 0.147 |
|  | p-value | 0.186 |
| right caudate | Rho Spearman | 0.225 |
|  | p-value | 0.041 |
| right MFG | Rho Spearman | 0.212 |
|  | p-value | 0.054 |
| right precentral gyrus | Rho Spearman | 0.156 |
|  | p-value | 0.159 |
| *SCZ and BD vs. HC* | | |
| right precentral gyrus | Rho Spearman | 0.439 |
|  | p-value | 0.18 |
| **ReHo values** | | |
| *SCZ vs. HC* |  |  |
| left SOG | Rho Spearman | 0.244 |
|  | p-value | 0.026 |
| right IOG | Rho Spearman | 0.133 |
|  | p-value | 0.229 |
| right postcentral gyrus | Rho Spearman | -0.007 |
|  | p-value | 0.949 |
| right postcentral gyrus | Rho Spearman | -0.043 |
|  | p-value | 0.703 |
| left cerebellum | Rho Spearman | 0 |
|  | p-value | 0.997 |
| left posterior orbital gyrus | Rho Spearman | 0.173 |
|  | p-value | 0.118 |
| left temporal pole | Rho Spearman | -0.081 |
|  | p-value | 0.469 |
| right anterior orbital gyrus | Rho Spearman | 0.05 |
|  | p-value | 0.654 |
| right hippocampus | Rho Spearman | -0.112 |
|  | p-value | 0.315 |
| right temporal pole | Rho Spearman | -0.15 |
|  | p-value | 0.175 |
| *BD vs. HC* | | |
| left PCC | Rho Spearman | -0.108 |
|  | p-value | 0.333 |
| right MTG | Rho Spearman | -0.12 |
|  | p-value | 0.28 |
| left anterior insula | Rho Spearman | 0.215 |
|  | p-value | 0.051 |
| right MFG | Rho Spearman | 0.254 |
|  | p-value | 0.02 |
| right STG | Rho Spearman | 0.228 |
|  | p-value | 0.038 |
| *SCZ vs. BD* | | |
| right calcarine scissure | Rho Spearman | 0.235 |
|  | p-value | 0.033 |
| *SCZ and BD vs. HC* | | |
| right IOG | Rho Spearman | 0.319 |
|  | p-value | 0.182 |

BD: bipolar disorder; DDD: daily doses of drug; HC: healthy controls; IFG: inferior frontal gyrus; IOG: inferior occipital gyrus; ITG: inferior temporal gyrus; MFG: middle frontal gyrus; MTG middle temporal gyrus; PCC: posterior cingulate cortex; SCZ: schizophrenia; SMG: supramarginal gyrus; SOG: superior occipital gyrus; SPL: superior parietal gyrus.

**Table S.6. Correlations between fALFF and ReHo values and antidepressants DDD**

| **fALFF and ReHo values** | **Statistics** | | **Antidepressants DDD** |
| --- | --- | --- | --- |
| **fALFF values** | | | |
| *SCZ vs. HC* | | | |
| left cuneus | Rho Spearman | | -0.129 |
|  | p-value | | 0.246 |
| left IOG | Rho Spearman | | -0.097 |
|  | p-value | | 0.381 |
| left MFG | Rho Spearman | | -0.302 |
|  | p-value | | 0.005 |
| left MFG | Rho Spearman | | -0.086 |
|  | p-value | | 0.442 |
| left postcentral gyrus | Rho Spearman | | -0.072 |
|  | p-value | | 0.515 |
| left SMG | Rho Spearman | | -0.13 |
|  | p-value | | 0.24 |
| left SPL | Rho Spearman | | -0.081 |
|  | p-value | | 0.465 |
| right precentral gyrus | Rho Spearman | | -0.005 |
|  | p-value | | 0.962 |
| right SPL | Rho Spearman | | -0.066 |
|  | p-value | | 0.555 |
| right postcentral gyrus | Rho Spearman | | -0.072 |
|  | p-value | | 0.518 |
| left caudate | Rho Spearman | | 0.238 |
|  | p-value | | 0.03 |
| left ITG | Rho Spearman | | 0.344 |
|  | p-value | | 0.001 |
| left ITG | Rho Spearman | | 0.206 |
|  | p-value | | 0.062 |
| right cerebellum | Rho Spearman | | 0.178 |
|  | p-value | | 0.108 |
| right PCC | Rho Spearman | | 0.273 |
|  | p-value | | 0.012 |
| right posterior insula | Rho Spearman | | 0.247 |
|  | p-value | | 0.025 |
| *BD vs. HC* | | | |
| right IOG | Rho Spearman | | -0.077 |
|  | p-value | | 0.488 |
| right middle postcentral gyrus | Rho Spearman | | 0.013 |
|  | p-value | | 0.906 |
| right occipital fusiform gyrus | Rho Spearman | | -0.023 |
|  | p-value | | 0.839 |
| left MFG | Rho Spearman | | 0.2 |
|  | p-value | | 0.069 |
| left thalamus | Rho Spearman | | 0.295 |
|  | p-value | | 0.007 |
| left transverse gyrus | Rho Spearman | | 0.211 |
|  | p-value | | 0.056 |
| right caudate | Rho Spearman | | 0.221 |
|  | p-value | | 0.044 |
| right MFG | Rho Spearman | | 0.171 |
|  | p-value | | 0.122 |
| right precentral gyrus | Rho Spearman | | 0.222 |
|  | p-value | | 0.044 |
| *SCZ and BD vs. HC* | | | |
| right precentral gyrus | Rho Spearman | 0.227 | |
|  | p-value | 0.093 | |
| **ReHo values** | | | |
| *SCZ vs. HC* | | | |
| left SOG | Rho Spearman | | -0.128 |
|  | p-value | | 0.248 |
| right IOG | Rho Spearman | | -0.177 |
|  | p-value | | 0.109 |
| right postcentral gyrus | Rho Spearman | | -0.251 |
|  | p-value | | 0.022 |
| right postcentral gyrus | Rho Spearman | | -0.116 |
|  | p-value | | 0.297 |
| left cerebellum | Rho Spearman | | 0.231 |
|  | p-value | | 0.036 |
| left posterior orbital gyrus | Rho Spearman | | -0.055 |
|  | p-value | | 0.621 |
| left temporal pole | Rho Spearman | | 0.159 |
|  | p-value | | 0.15 |
| right anterior orbital gyrus | Rho Spearman | | 0.071 |
|  | p-value | | 0.526 |
| right hippocampus | Rho Spearman | | 0.183 |
|  | p-value | | 0.098 |
| right temporal pole | Rho Spearman | | 0.009 |
|  | p-value | | 0.936 |
| *BD vs. HC* | | | |
| left PCC | Rho Spearman | | -0.009 |
|  | p-value | | 0.939 |
| right MTG | Rho Spearman | | -0.093 |
|  | p-value | | 0.403 |
| left anterior insula | Rho Spearman | | 0.111 |
|  | p-value | | 0.318 |
| right MFG | Rho Spearman | | 0.142 |
|  | p-value | | 0.201 |
| right STG | Rho Spearman | | 0.208 |
|  | p-value | | 0.059 |
| *SCZ vs. BD* | | | |
| right calcarine scissure | Rho Spearman | | -0.226 |
|  | p-value | | 0.04 |
| *SCZ and BD vs. HC* | | | |
| right IOG | Rho Spearman | | -0.117 |
|  | p-value | | 0.324 |

BD: bipolar disorder; DDD: daily doses of drug; HC: healthy controls; IFG: inferior frontal gyrus; IOG: inferior occipital gyrus; ITG: inferior temporal gyrus; MFG: middle frontal gyrus; MTG middle temporal gyrus; PCC: posterior cingulate cortex; SCZ: schizophrenia; SMG: supramarginal gyrus; SOG: superior occipital gyrus; SPL: superior parietal gyrus.

**Fig. S.1. Scatterplot of the correlations between cognitive components and clinical scales and fALFF and ReHo values in SCZ.**

**
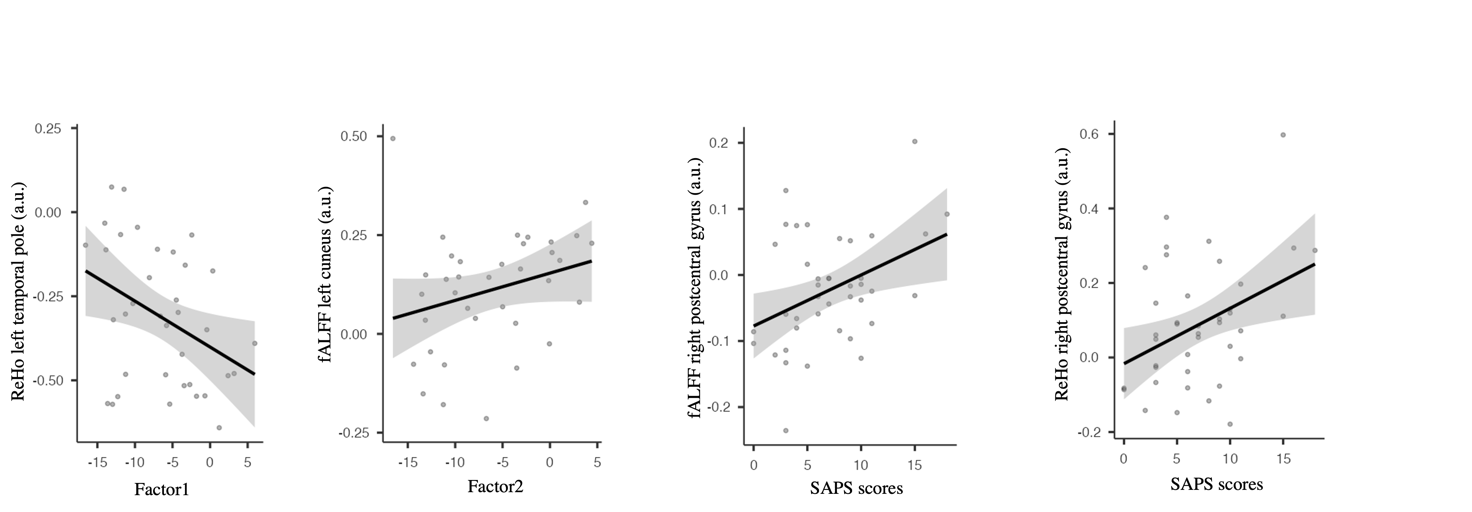
**

**Fig S.2. Scatterplot of the correlations between component 1 and fALFF and ReHo values in BD.**


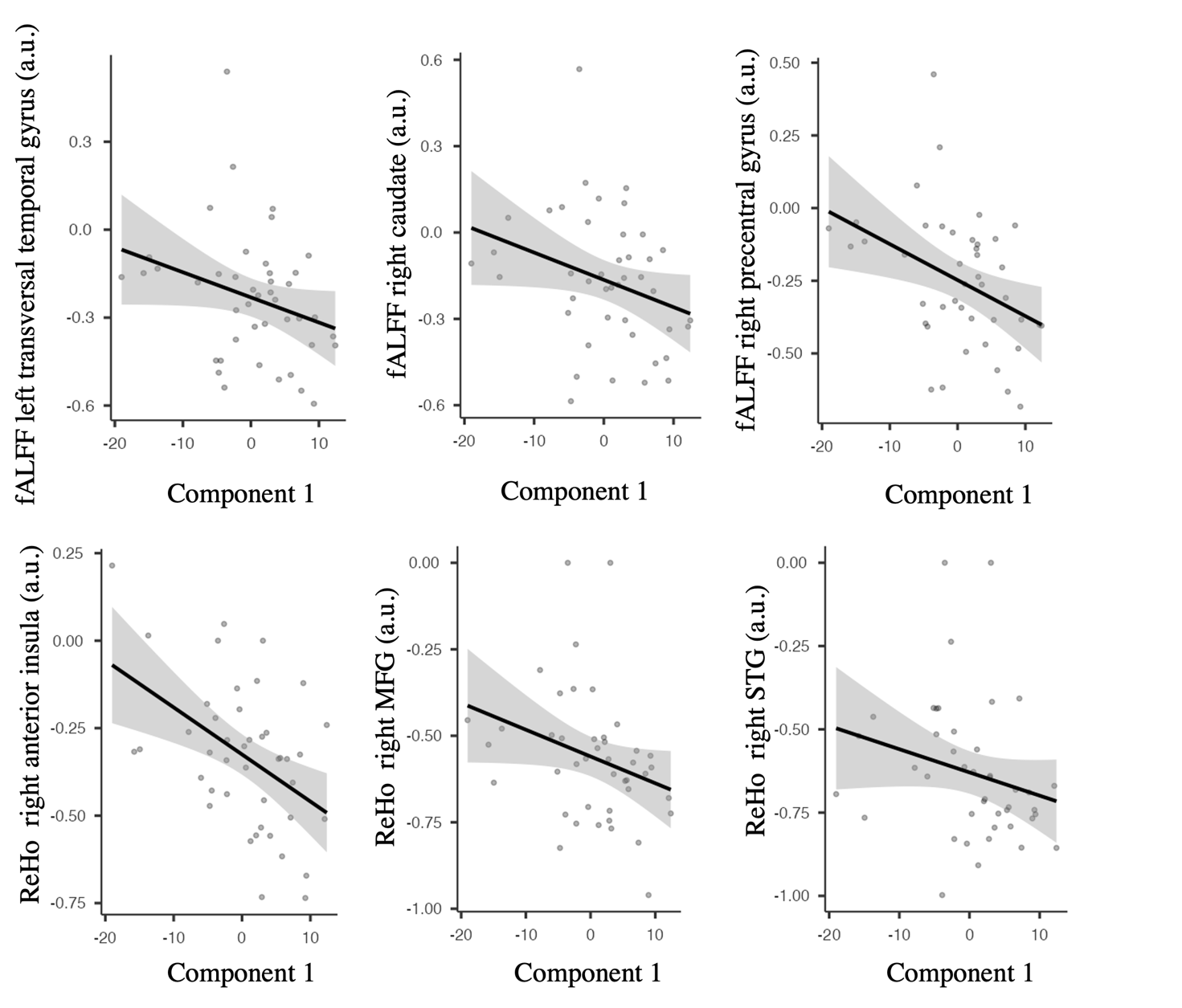


**Fig S.3. Scatterplot of the correlations between component 2 and fALFF and ReHo values in BD.**

**
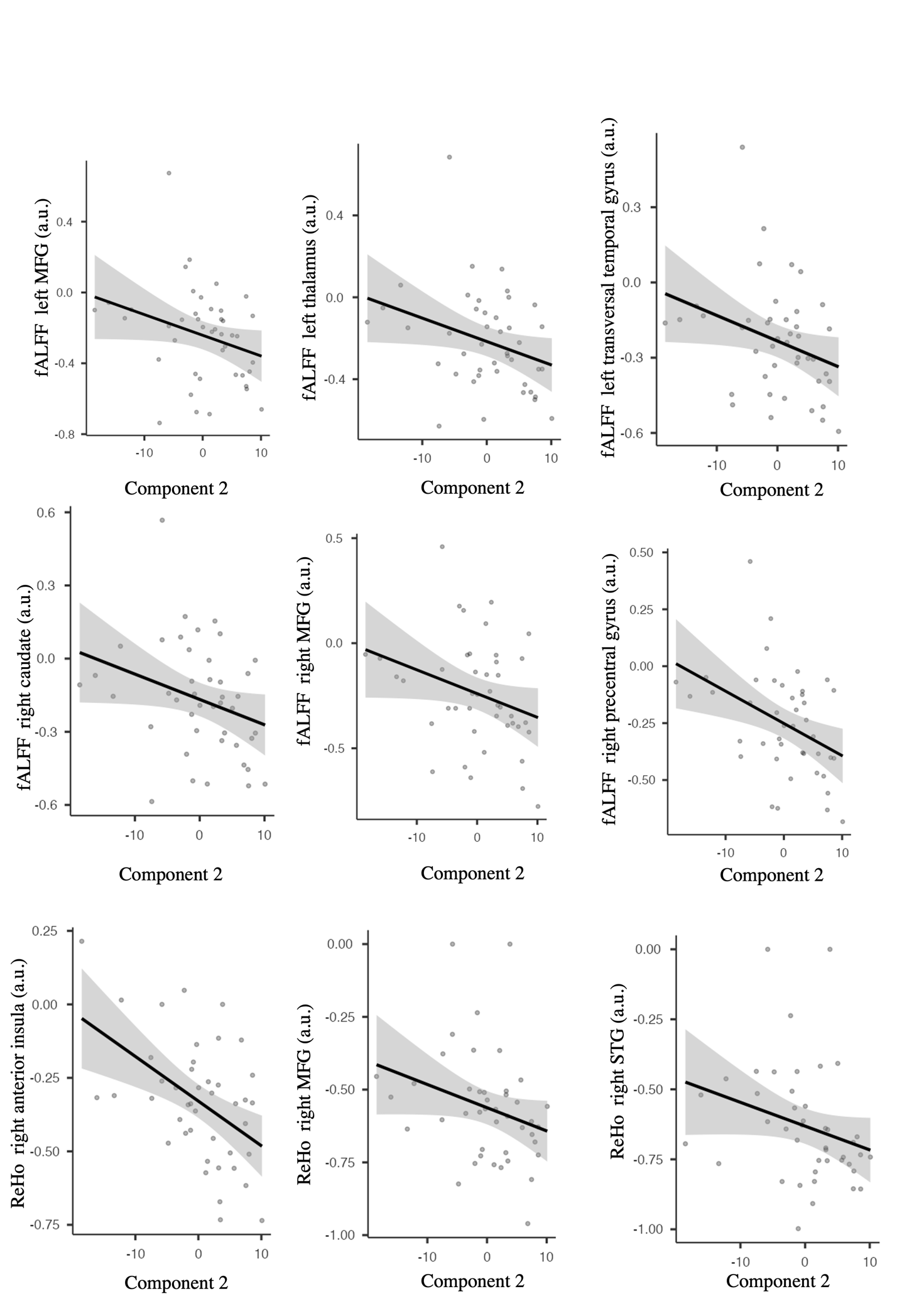
**

**Fig S.4. Scatterplot of the correlations between component 3 and fALFF and ReHo values in BD.**


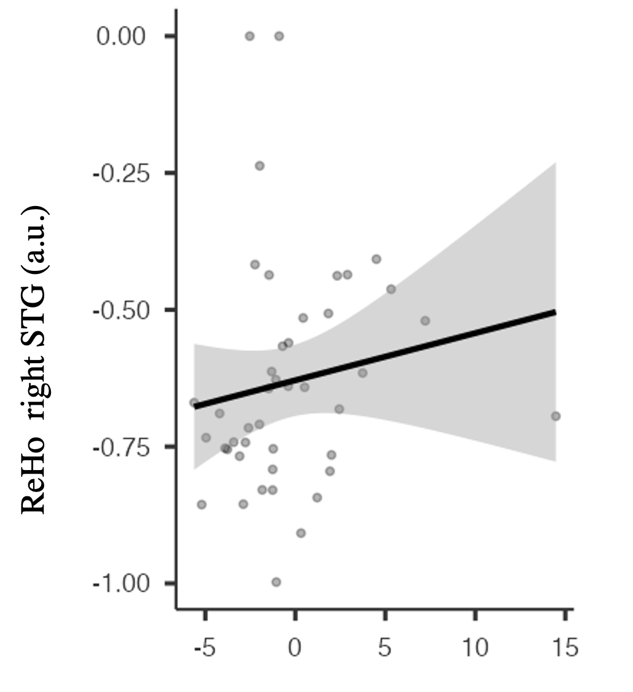

Supplement: Supplementary file 1 — Supplementary Material 1 [file 406_2024_1838_MOESM1_ESM.docx]
