# Supplementary material for: Diagnostic value of regional homogeneity and fractional amplitude of low-frequency fluctuations in the classification of schizophrenia and bipolar disorders
Source: Eur Arch Psychiatry Clin Neurosci. 2024 Jun 25;275(3):799–812. doi: 10.1007/s00406-024-01838-4 (PMC11947052; doi:10.1007/s00406-024-01838-4)
Supplement: Supplementary file 2 — Supplementary Material 2 [file 406_2024_1838_MOESM2_ESM.docx]

Padua, 26 April 2024

Prof. Falkai

Editor-in-Chief, European Archives of Psychiatry and Clinical Neuroscience

Dear Prof. Falkai,

enclosed for your consideration, it is a pleasure to submit for publication our revised manuscript entitled “**Diagnostic Value of Regional Homogeneity and Fractional Amplitude of Low-Frequency Fluctuations in the Classification of Schizophrenia and Bipolar Disorders”** to the European Archives of Psychiatry and Clinical Neuroscience.

We are grateful to the reviewer for the useful comments and suggestions that have helped us in improving our manuscript. In detail, we have provided a more thorough description of the sample, including details on clinical scales and pharmacological treatments. We have complemented our results with further analyses as suggested. We have also revised the discussion to better convey the meaning of the study. We believe that the results of our study will be of interest to the general readership of the European Archives of Psychiatry and Clinical Neuroscience.

The concerns of the reviewers have been addressed point by point in the rebuttal letter, including the responses and the changes made to the manuscript.

In response to the reviewers' requests to perform additional analyses and revisions to address specific concerns relative to machine learning approaches, Dr. Di Camillo’s workload significantly increased. Given the substantial contributions made by Dr. Di Camillo’s in response to reviewers' feedback, it became necessary to reevaluate the authorship order to accurately reflect the revised distribution of work and contributions. Therefore, Dr. Di Camillo has become the second author and Dr. Grimaldi has become the third author. The revised order of authorship was agreed upon among all authors regarding the distribution of credit based on the extent of each contribution to this research project.

All coauthors have reviewed and approved the contents and the authorship of the manuscript, and none of this material has been published or is being considered for publication elsewhere. Neither the coauthors nor we have any affiliation or involvement in any organization or entity with a direct financial interest in the subject matter or materials discussed in the manuscript.

Thank you for your consideration of our work.

With best wishes,

Fabio Sambataro, M.D., Ph.D.

Associate Professor of Psychiatry

Department of Neuroscience (DNS), University of Padova

Via Belzoni 160, 35121 Padua, Italy

Tel.: +39 0498211980; email: fabio.sambataro@unipd.it
